# Effect of exercise training before and after bariatric surgery: A systematic review and meta‐analysis

**DOI:** 10.1111/obr.13296

**Published:** 2021-06-03

**Authors:** Alice Bellicha, Marleen A. van Baak, Francesca Battista, Kristine Beaulieu, John E. Blundell, Luca Busetto, Eliana V. Carraça, Dror Dicker, Jorge Encantado, Andrea Ermolao, Nathalie Farpour‐Lambert, Adriyan Pramono, Euan Woodward, Jean‐Michel Oppert

**Affiliations:** ^1^ INSERM, Nutrition and Obesities: Systemic Approaches, NutriOmics Sorbonne University Paris France; ^2^ UFR SESS‐STAPS University Paris‐Est Créteil Créteil France; ^3^ NUTRIM School of Nutrition and Translational Research in Metabolism, Department of Human Biology Maastricht University Medical Centre+ Maastricht The Netherlands; ^4^ Sport and Exercise Medicine Division, Department of Medicine University of Padova Padua Italy; ^5^ Appetite Control and Energy Balance Group (ACEB), School of Psychology, Faculty of Medicine and Health University of Leeds Leeds UK; ^6^ Obesity Management Task Force (OMTF) European Association for the Study of obesity (EASO) London UK; ^7^ Department of Medicine University of Padova Padova Italy; ^8^ CIDEFES, Faculdade de Educação Física e Desport Universidade Lusófona de Humanidades e Tecnologias Lisbon Portugal; ^9^ Department of Internal Medicine, Hasharon Hospital, Rabin Medical Center, Sackler School of Medicine Tel Aviv University Tel Aviv Israel; ^10^ APPsyCI – Applied Psychology Research Center Capabilities & Inclusion ISPA ‐ University Institute Lisbon Portugal; ^11^ Obesity Prevention and Care Program Contrepoids, Service of Endocrinology, Diabetology, Nutrition and Patient Education, Department of Internal Medicine University Hospitals of Geneva and University of Geneva Geneva Switzerland; ^12^ Assistance Publique‐Hôpitaux de Paris (AP‐HP), Pitié‐Salpêtrière Hospital, Department of Nutrition, Institute of Cardiometabolism and Nutrition Sorbonne University Paris France

**Keywords:** bariatric surgery, body composition, exercise training, physical activity, severe obesity

## Abstract

We aimed to assess the effectiveness of exercise training programs in adults with severe obesity undergoing bariatric surgery. A systematic search of controlled trials published up to October 2019 that assigned participants to either a preoperative or postoperative exercise training group or a nonexercise group was performed. Meta‐analyses were conducted using random‐effects models. Twenty‐two training programs were assessed (18 performed after bariatric surgery). The effect of preoperative exercise training on postsurgery outcomes was reported in only one study. Compared with the control condition without exercise, postoperative exercise training led to higher weight loss (*N* = 14, mean difference [95% CI] = −1.8 [−3.2; −0.4] kg, *P* = 0.01), fat loss (*N* = 9, *P* = 0.01), increase in VO_2_max (*N* = 8, *P* < 0.0001), and increase in muscle strength (*N* = 9, *P* < 0.0001). No significant effect was found on lean body mass (*N* = 11). Preliminary evidence suggests a beneficial effect of postoperative exercise training on bone mineral density (*N* = 3, *P* < 0.001) and weight maintenance after the end of the intervention (*N* = 2, *P* < 0.001) but no significant effect on quality of life (*N* = 2), habitual physical activity (*N* = 2), or cardiometabolic outcomes (*N* < 4). In conclusion, exercise training performed after bariatric surgery improves physical fitness and leads to a small additional weight and fat loss and may prevent bone loss and weight regain after bariatric surgery.

## 1 INTRODUCTION

In patients with severe obesity, bariatric surgery produces marked and sustained weight loss; improves obesity comorbidities, physical function, and quality of life; and decreases mortality risk.^1^, ^2^, ^3^ To further enhance health benefits, lifestyle modifications including regular physical activity are recommended.^4^ However, most patients do not reach recommended levels of physical activity after bariatric surgery.^5^ According to recent reviews, only small increases in objectively assessed physical activity (e.g., using accelerometers) are observed 6–12 months after bariatric surgery,^3^, ^6^ suggesting the importance of physical activity promotion in these patients.

Over the past 10 years, a number of studies have assessed the effects of exercise training programs in the bariatric surgery setting, the majority of which have been performed after surgery. Contradictory findings were reported regarding weight loss, with two systematic reviews and meta‐analyses reporting greater weight loss in the exercise versus control group^7^, ^8^ and one review reporting no significant effect.^9^ The latter review,^9^ however, included two interventions based on respiratory muscle training,^10^, ^11^ a training modality that is not expected to impact weight loss. This highlights the importance of limiting inclusion criteria to whole‐body exercise training based on aerobic or resistance training, or both, when assessing the effect of exercise on weight loss. An increase in cardiorespiratory fitness^7^, ^12^ and in muscle strength^13^ has also been reported after a postoperative exercise training program, although the effect on muscle strength has not been assessed with a meta‐analysis. Similarly, the effects of exercise on important outcomes such as bone loss, quality of life, habitual physical activity, cardiometabolic outcomes, or weight loss maintenance after surgery have not been quantitatively synthetized in previous reviews.^7^, ^8^, ^9^, ^12^, ^13^ Finally, although there is only a limited number of studies assessing preoperative interventions, recent data on their effects have not been synthetized since 2015.^14^ Therefore, an updated systematic overview and meta‐analysis of this topic are relevant and needed.

In the context of the European Association for the Study of Obesity (EASO) Physical Activity Working Group (see summary paper for details), the aim of this systematic review was to examine the impact of physical activity interventions (i.e., exercise training programs) performed before or after bariatric surgery in subjects with obesity on weight loss, changes in body composition including bone mineral density, physical fitness, habitual physical activity, quality of life, and relevant health outcomes.

## 2 METHODS

This systematic review follows the Preferred Reporting Items for Systematic Reviews and Meta‐Analysis (PRISMA) guidelines and is registered in the International Prospective Register of Systematic Reviews (PROSPERO) database (registration number CRD42019157823).

### 2.1 Search strategy

Three electronic databases (PubMed, Web of Science, and EMBASE) were searched for original articles published up to October 2019 using the strategy “physical activity” AND “age” AND “bariatric surgery” (Table S1). Reference lists from the resulting reviews and articles were also screened to identify additional articles.

### 2.2 Study selection, inclusion, and exclusion

Articles were included if they involved adults (≥18 years) undergoing bariatric surgery (indicated when body mass index [BMI] ≥ 40 kg/m^2^ or ≥35 kg/m^2^ with at least one obesity comorbidity according to a majority of current guidelines) and participating in an exercise training program before or after surgery. Other inclusion criteria were (1) controlled trials with a comparison group of patients undergoing bariatric surgery receiving usual care without following an exercise training program; (2) exercise training based on aerobic and/or resistance and/or high‐intensity interval training (HIIT); (3) patients undergoing gastric bypass, sleeve gastrectomy, gastric banding, biliopancreatic diversion, or duodenal switch; and (4) preintervention to postintervention changes reported for at least one of the following outcome category: anthropometry or body composition, objectively measured physical activity or physical fitness, health‐related quality of life, and other relevant health outcomes. Presence of obesity comorbidities was not an exclusion criterion (see Section 2 of the summary paper for details). Abstracts and full texts were assessed for eligibility by one author (A. B.), and this selection was then checked by another author (J. M. O.). Any disagreement between reviewers was resolved through discussion.

### 2.3 Data extraction and synthesis

Data were extracted by one author (A. B.) using standardized forms and then checked by another author (J. M. O.). The characteristics of each included article included reference, study design, number of participants included, population characteristics (age, BMI, % female, and type of surgery), description of exercise intervention and comparison, outcomes, and duration of follow‐up. Additional data were obtained from six authors.^15^, ^16^, ^17^, ^18^, ^19^, ^20^

The findings pertaining to body weight and body composition (fat mass and lean body mass), physical fitness, habitual physical activity, health‐related quality of life, and health outcomes of each included article are reported. Data from intention‐to‐treat (ITT) analyses were included whenever reported in included studies. In addition, conclusions made by the study authors were reported, as well as our appreciation of the author's conclusion.

Effects of preoperative interventions were described using a semi‐quantitative approach because of a very limited number of studies (no more than three studies for a given outcome). The number of studies with positive, null, or negative findings is presented. Effects of postoperative interventions on changes in body weight, fat mass, lean body mass, VO_2_max, walking test distance, muscle strength, bone mineral density, moderate‐to‐vigorous physical activity (MVPA), quality of life, blood pressure, and metabolic outcomes (homeostatic model assessment of insulin resistance [HOMA‐IR], low‐density lipoprotein cholesterol (LDL‐c), high‐density lipoprotein cholesterol (HDL‐c), and triglycerides) were examined using random effects meta‐analyses (Review Manager version 5.3). The mean and standard deviation (SD) of absolute change in intervention and control groups were reported. Transformation methods were used for studies that did not provide the SD of absolute change but provided the exact *P* value for intragroup or intergroup analyses.^21^ Pooled‐effect estimates were expressed as the weighted mean difference (MD) between exercise and control groups for changes in body weight, body composition, bone mineral density, blood pressure, MVPA, and quality of life and as the weighted standardized mean difference (SMD) for changes in physical fitness and metabolic outcomes. The SMD for changes in VO_2_max was calculated based on changes in relative (mL/kg/min) or absolute (L/min) values of VO_2_max. The SMD for changes in walking test distance was calculated based on changes in the distance walked during different walking tests (i.e., 6‐min walk test and incremental shuttle walking test). The SMD for changes in muscle strength was calculated based on the changes in muscle strength assessed by various methods (i.e., lower‐limb or upper‐limb one‐repetition maximum and sit‐to‐stand test).

A *P* value <0.05 was considered statistically significant. Effect sizes were considered large, medium, small, and very small when SMD was >0.8, between 0.5 and 0.8, between 0.2 and 0.5, and below 0.2, respectively.^22^ Heterogeneity was assessed using *I* ^2^,^21^ with values interpreted as low at 25%, moderate at 50%, and high at 75%.^23^ Tau^2^ and test of homogeneity are also reported. Prediction intervals (PIs) were calculated when ≥10 studies were included in the meta‐analysis^21^ using the formula: 95%PI = MD ± 2 Tau^2^.^24^ To identify sources of heterogeneity, sensitivity analyses with the one‐study‐removed procedure were performed.^23^ Publication bias was assessed with visual inspection of the funnel plot and Egger's regression test when the number of studies included in the meta‐analysis was ≥10.

### 2.4 Quality assessment

Study quality was assessed with a standardized tool developed by the National Heart, Lung, and Blood Institute (NHLBI, USA) that has been previously used for defining guidelines for the management of obesity, including 14 criteria, as previously described.^25^ Three criteria were defined as “fatal flaws” when not met: (1) randomized study, (2) dropout rate < 20%, and (3) ITT analysis. Other criteria were adequate randomization method, treatment allocation concealment, blinding treatment assignment, blinding outcome assessors, similar baseline characteristics, differential dropout rate between groups <15%, high adherence (i.e., participation to exercise training sessions ≥70% or proportion of completers ≥70%), similar background treatments, valid and reliable outcome measures, sample size justification, and prespecified outcomes/subgroups. Study quality was defined as good, fair, and poor when 0, 1, or ≥2 fatal flaws were identified. Study quality was assessed by one author (A. B.), and this assessment was then checked by another author (J. M. O.). Any disagreement between the reviewers was resolved through discussion.

## 3 RESULTS

**FIGURE 1:**
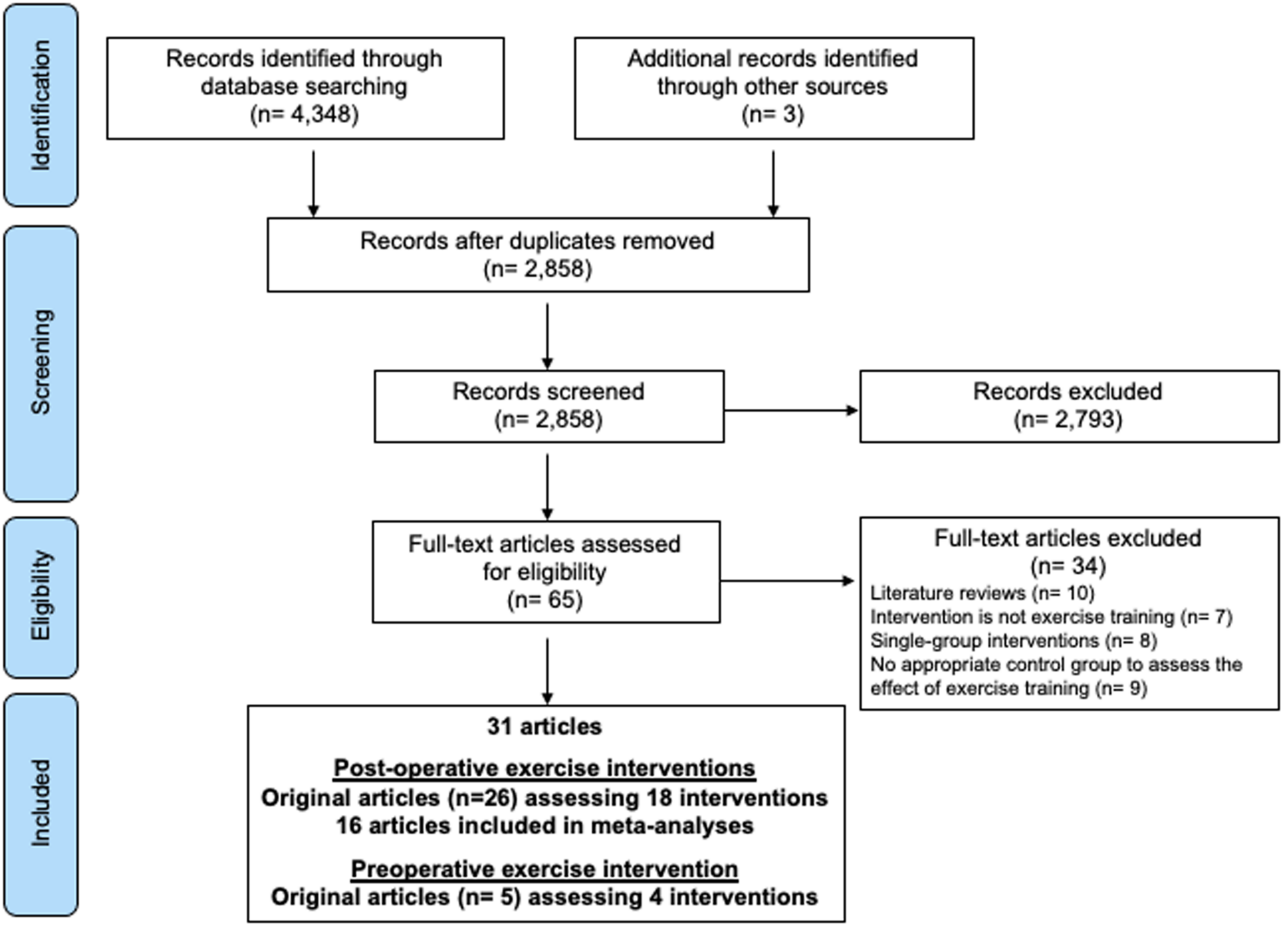
Systematic review flow diagram

The database search yielded 4348 articles (2858 after removing duplicates), 2793 of which were eliminated based on titles and abstracts alone (Figure 1). The full text was retrieved from 65 articles, and 31 met the inclusion criteria. Parent trials by Coen et al.^26^ and Mundbjerg et al.^27^ were each reported in four additional articles^28^, ^29^, ^30^, ^31^, ^32^, ^33^, ^34^, ^35^; those by Castello et al.^36^ and by Baillot et al.^37^ were each reported in one additional article.^38^, ^39^ One article assessed two distinct interventions.^17^ Therefore, a total of 22 distinct interventions were included of which 16 were included in the meta‐analyses.

### 3.1 Study characteristics

Studies were published between 2011 and 2019 and were either randomized (*n* = 14, 67%) or nonrandomized (*n* = 7, 33%)^16^, ^18^, ^20^, ^40^, ^41^, ^42^, ^43^ controlled trials (Table 1). The median (min–max) total sample size was 33 (6–220). Median age and BMI at baseline were 41 (33–54) years and 43.1 (29.6–50.8) kg/m^2^. Both males and females were included in 15 studies of which the median percentage of females was 83 (60–92)%. Six studies included females only.^18^, ^36^, ^40^, ^44^, ^45^, ^46^

**TABLE 1:** Characteristics of included controlled trials

Reference	Study design Surgery type	Population	Intervention	Comparison	Outcomes	Follow‐up duration
Baillot et al.^37^	RCT Not reported	Exercise group: *N* = 15 Age: 41.4 (38.3–54.3) years BMI: 44.8 (42.1–53.0) kg/m^2^ Female: 80% Control group: *N* = 14 Age: 43.3 (36.5–47.1) years BMI: 47.8 (40.3–54.0) kg/m^2^ Female: 79%	Preoperative intervention ‐ Program duration: 3 months ‐ Aerobic + resistance training ‐ 3 sessions/week: 30 min of aerobic exercise (55–85% RHR) + 2–3 series of 12–15 rep on 9 resistance exercises ‐ Supervision: total (by PA specialists)	Usual care	‐ Body weight ‐ Body composition ‐ VO_2_peak ‐ Walking test (6MWT) ‐ Quality of life ‐ Blood pressure	Postintervention
Baillot et al.^38^	RCT RYGB, SG	‐‐	Same intervention as Baillot et al.^37^		‐ Body weight ‐ Body composition ‐ VO_2_peak ‐ Walking test (6MWT) ‐ Accelerometer‐assessed PA ‐ Quality of life ‐ Blood pressure	Long term
Campanha‐Versiani et al.^16^	Non‐RCT RYGB	Exercise group: *N* = 18 Age: 37.2 (9.3) years BMI: 42.5 (4.0) kg/m^2^ Female: 83% Control group: *N* = 19 Age: 37.0 (10.8) years BMI: 41.7 (4.6) kg/m^2^ Female: 83%	Postoperative intervention ‐ Program duration: 9 months ‐ Start: 3 months after surgery ‐ Aerobic + resistance training ‐ 2 sessions/week: 1–3 series of 10–12 rep on 8 resistance exercises + 25 min of aerobic exercise (70–80% RHR) ‐ Supervision: total (staff not reported)	Usual care	‐ Body weight ‐ Body composition ‐ Bone mineral density ‐ Muscle strength	Postintervention
Castello et al.^36^	RCT RYGB	Exercise group: *N* = 21 Age: 38.0 (4.0) years BMI: 45.6 (1.5) kg/m^2^ Female: 100% Control group: *N* = 19 Age: 36.0 (4.0) years BMI: 44.5 (1.0) kg/m^2^ Female: 100%	Postoperative intervention ‐ Program duration: 3 months ‐ Start: 1 month after surgery ‐ Aerobic training ‐ 3 sessions/week: 40 min of aerobic exercise (50–70% HR_max_) ‐ Supervision: total (by physiotherapists)	Usual care	‐ Body weight ‐ Waist circumference ‐ Body composition ‐ Walking test (6MWT)	Postintervention
Castello‐Simoes et al.^39^	‐‐	‐‐	Same intervention as Castello et al.^36^		‐ BMI ‐ Lung function	Postintervention
Coen et al.^26^	RCT RYGB	Exercise group: *N* = 66 Age: 41.3 (9.7) years BMI: 38.8 (6.1) kg/m^2^ Female: 89% Control group: *N* = 62 Age: 41.9 (10.3) years BMI: 38.3 (6.9) kg/m^2^ Female: 94%	Postoperative intervention ‐ Program duration: 6 months ‐ Start: 1 month after surgery ‐ Aerobic training ‐ 3–5 sessions/week: 120 min of aerobic exercise (60–70% HR_max_) per week ‐ Supervision: partial (by trained exercise physiologist)	Usual care	‐ Body weight ‐ Waist circumference ‐ Body composition ‐ VO_2_peak ‐ Glucose metabolism ‐ Lipid profile ‐ Blood pressure	Postintervention
Coen et al.^28^	‐‐	‐‐	Same intervention as Coen et al.^26^		‐ VO_2_peak	Postintervention
Woodlief et al.^29^	‐‐	‐‐	Same intervention as Coen et al.^26^		‐ Resting metabolic rate	Postintervention
Carnero et al.^30^	‐‐	‐‐	Same intervention as Coen et al.^26^		‐ Accelerometry‐assessed PA	Postintervention
Nunez Lopez et al.^31^	‐‐	‐‐	Same intervention as Coen et al.^26^		‐ Bone mass	Postintervention
Coleman et al.^15^	RCT RYGB, SG, lap band	Exercise group: *N* = 26 Age: 52.0 (10.9) years BMI: 45.0 (7.6) kg/m^2^ Female: 85% Control group: *N* = 25 Age: 46.6 (12.0) years BMI: 44.5 (5.5) kg/m^2^ Female: 100%	Postoperative intervention ‐ Program duration: 6 months ‐ Start: 6–24 months after surgery ‐ Aerobic + resistance training ‐ 2 sessions/week: 60 min (details not reported) ‐ Supervision: partial (staff not reported)	Usual care	‐ Body weight ‐ Pedometer‐assessed PA ‐ Muscle strength ‐ Walking test (6MWT)	Intermediate term
Daniels et al.^48^	RCT RYGB	Exercise group: *N* = 8 Age: not reported BMI: not reported Female: 85% Control group: *N* = 8 Age: not reported BMI: not reported Female: 100% All participants Age: 44.9 (10.2) years	Postoperative intervention ‐ Program duration: 3 months ‐ Start: 2 months after surgery ‐ Resistance training ‐ 3 sessions/week: 1–4 sets of 8–15 rep on 8–10 resistance exercises ‐ Supervision: not reported	Usual care	‐ Body weight ‐ Body composition ‐ Skeletal muscle mass ‐ Muscle strength	Postintervention
Hassannejad et al.^17^	RCT RYGB, SG	Exercise (aerobic) group: *N* = 20 Age: 33.3 (8.4) years BMI: 47.9 (6.7) kg/m^2^ Female: 75% Exercise (aerobic + resistance) group: *N* = 20 Age: 35.4 (8.1) years BMI: 42.9 (3.9) kg/m^2^ Female: 70% Control group: *N* = 20 Age: 36.7 (6.2) years BMI: 46.6 (6.0) kg/m^2^ Female: 80%	Postoperative intervention ‐ Program duration: 3 months ‐ Start: immediately (aerobic) and 5‐week (aerobic + resistance) after surgery ‐ Aerobic training or aerobic + resistance training ‐ 3–5 sessions/week: 150–200 min of aerobic exercise (12–14 on Borg scale) per week + 20–30 min of resistance exercise (only in the aerobic + resistance group) ‐ Supervision: none	Usual care	‐ Body weight ‐ Body composition ‐ Skeletal muscle mass ‐ Muscle strength ‐ Walking test	Postintervention
Herring et al.^49^	RCT RYGB, SG, GB	Exercise group: *N* = 12 Age: 44.3 (7.9) years BMI: 38.2 (6.1) kg/m^2^ Female: 92% Control group: *N* = 12 Age: 52.4 (8.1) years BMI: 39.4 (4.3) kg/m^2^ Female: 92%	Postoperative intervention ‐ Program duration: 3 months ‐ Start: 12–24 months after surgery ‐ Aerobic + resistance training ‐ 3 sessions/week: 45 min of aerobic exercise (64–77% HR_max_) + 3 sets of 12 rep on 4 resistance exercises ‐ Supervision: total (by qualified gym instructors)	Usual care	‐ Body weight ‐ Waist circumference ‐ Body composition ‐ Accelerometry‐assessed PA ‐ Muscle strength ‐ Walking test ‐ Blood pressure	Short term
Huck^20^	Non‐RCT RYGB, GB	Exercise group: *N* = 7 Age: 53.6 (8.2) years BMI: 37.7 (6.3) kg/m^2^ Female: 86% Control group: *N* = 8 Age: 44.0 (9.7) years BMI: 32.7 (4.2) kg/m^2^ Female: 75%	Postoperative intervention ‐ Program duration: 3 months ‐ Start: 4 months after surgery ‐ Resistance training ‐ 2–3 sessions/week: 1–3 sets of 8–12 rep on 8–10 resistance exercises ‐ Supervision: total (by certified strength and conditioning specialist)	Usual care	‐ Body weight ‐ Waist circumference ‐ Body composition ‐ VO_2_peak ‐ Muscle strength	Postintervention
Marchesi et al.^40^	Non‐RCT RYGB	Exercise group: *N* = 10 Age: 43.1 (37–48) years BMI: 29.6 (23.9–33.6) kg/m^2^ Female: 100% Control group: *N* = 10 Age: 39.1 (31–46) years BMI: 30.1 (25.9–39.3) kg/m^2^ Female: 100%	Postoperative intervention ‐ Program duration: 10 months ‐ Start: 1–3 years after surgery ‐ Aerobic training ‐ 3 sessions/week: 60 min of aerobic exercise (55–65% HR_max_ with some sessions at 65–85% HR_max_) ‐ Supervision: total (by personal trainers)	Usual care	‐ Body weight ‐ Waist circumference ‐ Body composition ‐ VO_2_peak ‐ Quality of life ‐ Glucose metabolism ‐ Lipid profile	Postintervention
Marc‐Hernandez et al.^41^ ^(p)^	Non‐RCT Not reported	Exercise group: *N* = 10 Age: 42.5 (5.1) years BMI: 47.5 (7.1) kg/m^2^ Female: 70% Exercise group: *N* = 8 Age: 37.5 (10.3) years BMI: 41.5 (2.7) kg/m^2^ Female: 100%	Preoperative intervention ‐ Program duration: 3 months ‐ Aerobic (including HIIT) + resistance training ‐ 2–4 sessions/week: 35–50 min of aerobic exercise (60–70% HR_max_) or 20 min HIIT (60–80% HR_max_) + 1–4 sets of 15–20 rep on 4–7 resistance exercises ‐ Supervision: total (staff not reported)	Usual care	‐ Body weight ‐ Body composition ‐ VO_2_peak ‐ Muscle strength ‐ Lipid profile ‐ Quality of life	Postintervention
Marcon et al.^47^	RCT Not reported	Exercise group: *N* = 22 Age: 43.4 (2.3) years BMI: 50.8 (9.6) kg/m^2^ Female: 82% Exercise + counseling group: *N* = 17 Age: 50.1 (2.8) years BMI: 45 (4.1) kg/m^2^ Female: 100% Control group: *N* = 18 Age: 42.5 (2.7) years BMI: 47.1 (7.6) Female: 89%	Preoperative intervention ‐ Program duration: 4 months ‐ Aerobic training or aerobic training + cognitive‐behavioral therapy ‐ 2 sessions/week: 25 min of aerobic exercise (intensity not reported) ‐ Supervision: total (by personal trainers)	Usual care	‐ Body weight ‐ Walking test (6MWT) ‐ Estimated VO_2_peak ‐ Glucose metabolism ‐ Blood pressure ‐ Lipid profile	Postintervention
Mundbjerg et al.^27^	RCT RYGB	Exercise group: *N* = 32 Age: 42.3 (9.4) years BMI: 43.1 (6.7) kg/m^2^ Female: 66% Control group: *N* = 28 Age: 42.4 (9.0) years BMI: 42.8 (5.5) kg/m^2^ Female: 75%	Postoperative intervention ‐ Program duration: 6 months ‐ Start: 6 months after surgery ‐ Aerobic + resistance training ‐ 2 sessions/week: 30 min of aerobic exercise (15–17 on the Borg scale) + 10 min of resistance exercise Supervision: total (by physiotherapists)	Usual care	‐ Body weight ‐ Waist circumference ‐ Abdominal fat volume ‐ Blood pressure ‐ Resting heart rate ‐ Glucose metabolism ‐ Lipid profile	Long term
Mundbjerg et al.^32^	‐‐	‐‐	Same intervention as Mundbjerg et al.^27^		‐ VO_2_peak ‐ Muscle strength	Long‐term
Stolberg et al.^34^	‐‐	‐‐	Same intervention as Mundbjerg et al.^27^		‐ Inflammation ‐ Endothelial function	Long‐term
Stolberg et al.^33^	‐‐	‐‐	Same intervention as Mundbjerg et al.^27^		‐ Accelerometer‐assessed PA ‐ Quality of life	Long‐term
Stolberg et al.^35^	‐‐	‐‐	Same intervention as Mundbjerg et al.^27^		‐ Markers of coagulation	Long‐term
Murai et al.^44^	RCT RYGB	Exercise group: *N* = 31 Age: 40.0 (7.8) years BMI: 49.8 (7.0) kg/m^2^ Female: 100% Control group: *N* = 32 Age: 42.1 (8.2) years BMI: 48.5 (8.1) kg/m^2^ Female: 100%	Postoperative intervention ‐ Program duration: 6 months ‐ Start: 3 months after surgery ‐ Aerobic + resistance training ‐ 2 sessions/week: 30–60 min of moderate‐intensity aerobic exercise + 3 sets of 8–12 rep on 7 resistance exercises ‐ Supervision: total (staff not reported)	Usual care	‐ Bone mineral density	Postintervention
Muschitz et al.^50^	RCT RYGB, SG	Exercise group: *N* = 110 Age: 41.0 (34;0; 45.0) years BMI: 44.3 (41.1; 47.9) kg/m^2^ Female: 60% Control group: *N* = 110 Age: 40.0 (35.0; 45.8) years BMI: 44.2 (40.7; 47.7) kg/m^2^ Female: 56%	Post‐operative intervention ‐ Program duration: 24 months Start: 2 weeks after surgery ‐ Aerobic + resistance training + protein, calcium and vit. D supplementation ‐ 5 sessions/week of aerobic training (45 min, intensity not reported) + 2 sessions/week of resistance training (30 min, intensity not reported) ‐ Supervision: none	Usual care	‐ Body weight ‐ Body composition ‐ Bone mineral density ‐ Quality of life	Postintervention
Onofre et al.^18^	Non‐RCT RYGB, SG	Exercise group: *N* = 6 Age: 40.3 (10.7) years BMI: 46.1 (7.0) kg/m^2^ Female: 100% Control group: *N* = 6 Age: 39.5 (7.2) years BMI: 44.9 (9.0) kg/m^2^ Female: 100%	Postoperative intervention ‐ Program duration: 3 months ‐ Start: 3 months after surgery ‐ Aerobic + resistance training ‐ 3 sessions/week: 30 min of aerobic exercise (40–60% RHR with high‐intensity periods at 85–90% RHR) + 20 min of resistance exercise ‐ Supervision: total (by physiotherapists)	Usual care	‐ Body weight ‐ Waist circumference ‐ VO_2_peak	Postintervention
Oppert et al.^45^	RCT RYGB	Exercise + protein supplementation group: *N* = 23 Age: 40.9 (10.8) years BMI: 45.2 (5.2) kg/m^2^ Female: 100% Protein supplementation group: *N* = 31 Age: 42.5 (8.7) years BMI: 43.3 (6.0) kg/m^2^ Female: 100% Control group: *N* = 22 Age: 43.9 (10.7) years BMI: 43.6 (6.2) kg/m^2^ Female: 100%	Postoperative intervention ‐ Program duration: 6 months ‐ Start: 1.5 months after surgery ‐ Resistance training ‐ 3 sessions/week: 4 sets of 8–12 rep on 6 resistance exercises ‐ Supervision: total (by trained PA instructors)	Usual care	‐ Body weight ‐ Body composition ‐ Accelerometry‐assessed PA ‐ VO_2_peak ‐ Muscle strength ‐ Quality of life ‐ Dietary (protein) intake	Postintervention
Pico‐Sirvent et al.^42^	Non‐RCT Not reported	Exercise group: *N* = 3 Age: 39.7 (10.2) years BMI: 38.0 (1.2) kg/m^2^ Female: 67% Control group: *N* = 3 Age: 36.7 (0.6) years BMI: 29.5 (0.6) kg/m^2^ Female: 100%	Preoperative intervention ‐ Program duration: 6 months ‐ Aerobic (including HIIT) + resistance training ‐ 2 sessions/week of 50 min of aerobic exercise (60–85% HR_max_) + 2 sessions/week of HIIT (30″/30″ at 95% HR_max_) + 1–4 series of 10–20 rep on 5 resistance exercises ‐ Supervision: total (staff not reported)	Usual care	‐ Body weight ‐ Body composition ‐ VO_2_peak ‐ Muscle strength	Postintervention
Rojhani‐Shirazi et al.^46^	RCT SG	Exercise group: *N* = 16 Age: 36.1 (6.7) years BMI: 40.5 (5.4) kg/m^2^ Female: 100% Control group: *N* = 16 Age: 36.6 (7.8) years BMI: 44.0 (7.2) kg/m^2^ Female: 100%	Postoperative intervention ‐ Program duration: 1 month ‐ Start: 5 days after surgery ‐ Balance training ‐ 4 sessions/week of 30–45 min ‐ Supervision: Not reported	Usual care	‐ Body weight ‐ Waist circumference ‐ Balance control	Postintervention
Shah et al.^19^	RCT RYGB, GB	Exercise group: *N* = 21 Age: 47.3 (10.0) years BMI: 42.4 (6.9) kg/m^2^ Female: 90% Control group: *N* = 12 Age: 53.9 (8.8) years BMI: 41.0 (3.7) kg/m^2^ Female: 92%	Postoperative intervention ‐ Program duration: 3 months ‐ Start: 3–42 months after surgery ‐ Aerobic training ‐ 5 sessions/week: aerobic exercise (60–70% VO_2_max) resulting in an energy expenditure ≥ 2000 kcal/week ‐ Supervision: total (by a study investigator)	Usual care	‐ Body weight ‐ Waist circumference ‐ Body composition ‐ Pedometer‐assessed PA ‐ VO_2_max ‐ Glucose metabolism ‐ Lipid profile ‐ Quality of life	Postintervention
Stegen et al.^43^	Non‐RCT RYGB	Exercise group: *N* = 8 Age: 39.9 (9.9) years BMI: 45.3 (2.7) kg/m^2^ Female: 88% Control group: *N* = 7 Age: 43.1 (5.6) years BMI: 40.4 (8.1) kg/m^2^ Female: 57%	Postoperative intervention ‐ Program duration: 3 months ‐ Start: 1 month after surgery ‐ Aerobic + resistance training ‐ 3 sessions/week: 25 min of resistance exercise (1–3 sets of 10 rep) + 30 min of aerobic exercise (60% HR_max_) ‐ Supervision: total (by sports science students)	Usual care	‐ Body weight ‐ Waist circumference ‐ Body composition ‐ VO_2_peak ‐ Muscle strength ‐ Walking test (6MWT)	Postintervention

*Note:* Articles are presented in alphabetical order, and articles reporting results from the same trial are presented together. Assessment performed immediately after the intervention, less than 6 months after the intervention, 6–12 months after the intervention, or more than 12 months after the intervention was referred to as postintervention, short term, intermediate term, and long term, respectively.

Abbreviations: GB, gastric banding; HR, heart rate; non‐RCT, nonrandomized controlled trial; PA, physical activity; RCT, randomized controlled trial; RHR, reserve heart rate; RYGB, Roux‐en‐Y gastric bypass; SG, sleeve gastrectomy; 6MWT, 6‐min walk test.

Interventions were performed presurgery in four (19%) studies^37^, ^41^, ^42^, ^47^ and postsurgery in 17 (81%) studies. In the latter studies, patients underwent Roux‐en‐Y gastric bypass (RYGB) in nine studies,^16^, ^26^, ^27^, ^36^, ^40^, ^43^, ^44^, ^45^, ^48^ sleeve gastrectomy in one study,^46^ and either RYGB, gastric banding, or sleeve gastrectomy in the remaining studies.^15^, ^17^, ^18^, ^19^, ^20^, ^49^, ^50^ Exercise training started 2 months (1 week–24 months) postsurgery, with a duration of 3 (1–24) months and 3 (2–5) sessions per week. In four studies,^15^, ^19^, ^40^, ^49^ exercise training started during the second postoperative year and inclusion spread over several months. Aerobic training was performed in six (27%) studies,^17^, ^19^, ^26^, ^36^, ^40^, ^47^ resistance training in three (14%) studies,^20^, ^45^, ^48^ a combination of aerobic and resistance training in 12 (54%) studies,^15^, ^16^, ^17^, ^18^, ^27^, ^37^, ^41^, ^42^, ^43^, ^44^, ^49^, ^50^ and balance training in one (5%) study.^46^ In two preoperative programs, aerobic training incorporated periods of HIIT.^41^, ^42^ Two studies included protein supplementation in the exercise group.^45^, ^50^ Exercise sessions were fully supervised in 15 (71%) studies, partially supervised in two studies^15^, ^26^ or not supervised in two studies.^17^, ^50^ Supervision was not reported in two studies.^46^, ^48^

The most frequently reported outcomes were body weight (*n* = 22, 100%), physical fitness (*n* = 20, 91%), body composition (*n* = 18, 82%), health‐related quality of life (*n* = 8, 36%), and accelerometer‐ or pedometer‐assessed physical activity (*n* = 7, 32%). Follow‐up assessments were performed immediately after the intervention (i.e., postintervention, in 18 [82%] studies), at short term (<6 months) in one study,^49^ at intermediate term (6–12 months) in one study,^15^ and at long term (>12 months) in two studies.^27^, ^38^ Findings of all included studies are presented in Table S2.

### 3.2 Preoperative interventions

Because of the very limited number of studies available,^37^, ^38^, ^41^, ^47^ we were not able to perform meta‐analyses to assess the effect of preoperative interventions. Instead, a semi‐quantitative analysis is presented. At the postintervention follow‐up (presurgery), walking test distance increased in the exercise group compared with the control group in two (100%) studies,^37^, ^47^ and body weight decreased in two (67%) studies^41^, ^47^ (Table 2). Fat mass, muscle strength, quality of life, glucose metabolism, and lipid profile were improved in one (50%) study.^41^, ^47^ Nonsignificant findings were found for changes in VO_2_max in the exercise versus control group in two (67%) studies,^37^, ^41^ in blood pressure in two (67%) studies,^37^, ^47^ and in lean body mass in one (100%) study.^41^ Long‐term follow‐up (at 1‐year postsurgery) was assessed in only one study, reporting a greater increase in habitual physical activity in the exercise versus control group.^38^ BMI and lean body mass loss were also larger in the exercise group, but no difference was found for changes in fat mass, physical fitness, and quality of life.

**TABLE 2:** Effectiveness of preoperative exercise training programs

Reference	Body weight BMI	Fat mass % body fat	LBM	VO_2_max	Walking test	Muscle strength	Habitual PA	HRQOL	Blood pressure	Glucose metabolism	Lipid profile
Postintervention (before bariatric surgery)											
Baillot et al.^37^	ns	ns		ns	(+)	(+)		ns	ns		
Marcon et al.^47^	(+)			(+)	(+)				ns	(+)	(+)
Marc‐Hernandez et al.^41^	(+)	(+)	ns	ns		ns		(+)	(+)	ns	ns
Long‐term follow‐up (after bariatric surgery)											
Baillot et al.^38^	(+)	ns	(−)	ns	(+)	ns	(+)	ns	ns		

*Note:* Glucose metabolism was assessed by fasting glucose or HbA1c, lipid profile by LDL‐c, HDLc, and triglycerides. The study by Pico‐Sirvent et al.^42^ was not included because no statistical analysis was performed (three participants included in each group).

Abbreviations: BMD, bone mineral density; HRQOL, health‐related quality of life; LBM, lean body mass; ns, no significant difference in the exercise group compared to the control group; PA, physical activity; (+), significant improvement in the exercise group compared with the control group; (−), significant deterioration in the exercise group compared with the control group.

### 3.3 Postoperative interventions

Findings of meta‐analyses are summarized in Table 3.

**TABLE 3:** Summary of findings of meta‐analyses

Outcome	*N* studies	MD [95% CI] or SMD [95% CI]	*P* value	*I*^2^ ‐ Tau^2^ (*P* value)	[95% PI]
Effect observed after the intervention					
Change in body weight	14	MD: −1.8 [−3.2; −0.4] kg	0.01	35% ‐ 2.28 (0.09)	[−5.4; 1.8]
Change in fat mass	9	MD: −2.1 [−3.7; −0.5] kg	0.01	50% ‐ 2.76 (0.04)	
Change in lean body mass	11	MD: 0.7 [−0.2; 1.6] kg	0.13	45% ‐ 0.92 (0.05)	[−1.7; 3.1]
Change in bone mineral density	3	SMD: 0.44 [0.21; 0.67]	0.0002	0% ‐ 0.0 (0.40)	
Change in VO_2_max	8	SMD: 0.70 [0.35; 1.10]	<0.0001	42% ‐ 0.10 (0.10)	
Change in muscle strength	9	SMD: 0.82 [0.48; 1.16]	<0.0001	42% ‐ 0.11 (0.09)	
Change in walking distance	6	SMD: 1.46 [0.27; 2.66]	0.02	90% ‐ 1.98 (<0.001)	
Systolic blood pressure	4	MD: −4.2 [−9.3; 1.0] mmHg	0.12	47% ‐ 12.7 (0.13)	
Diastolic blood pressure	4	MD: −2.3 [−8.5; 3.9] mmHg	0.47	77% ‐ 29.1 (0.005)	
HOMA‐IR	2	SMD: 0.14 [−0.10; 0.38]	0.27	0% ‐ 0.0 (0.49)	
LDL‐c	3	SMD: −0.18 [−0.46; 0.09]	0.20	0% ‐ 0.0 (0.59)	
HDL‐c	4	SMD: 0.10 [−0.16; 0.37]	0.45	0% ‐ 0.0 (0.51)	
Triglycerides	4	SMD: 0.01 [−0.26; 0.27]	0.97	0% ‐ 0.0 (0.88)	
Quality of life—physical dimension	2	MD: −2.5 [−5.1; 0.2]	0.07	0% ‐ 0.0 (0.32)	
Quality of life—mental dimension	2	MD: 3.9 [−0.5; 8.3]	0.08	0% ‐ 0.0 (0.37)	
Effect observed after a follow‐up without exercise^a^					
Change in body weight	2	MD: −4.7 [−7.2; −2.1] kg	0.0003	0% ‐ 0.0 (0.49)	
Change in muscle strength	2	SMD: 0.78 [−0.08; 1.64]	0.08	57% ‐ 0.23 (0.13)	

*Note*: 95% PI: 95% prediction intervals. 95% PI were calculated when the number of studies included in the meta‐analysis was ≥10 for a given outcome.

Abbreviations: HDL‐c, high‐density lipoprotein cholesterol; HOMA‐IR, homeostatic model assessment of insulin resistance; LDL‐c, low‐density lipoprotein cholesterol; MD, mean difference; PI, prediction interval; SMD, standardized mean difference.

^a^ Data are the difference between body weight or muscle strength after several months of follow‐up without exercise (3 months in the study by Herring et al.^49^ and 12 months in the study by Mundbjerg et al.^27^) versus same outcomes measured before the beginning of the exercise training program.

#### 3.3.1 Body composition

**FIGURE 2:**
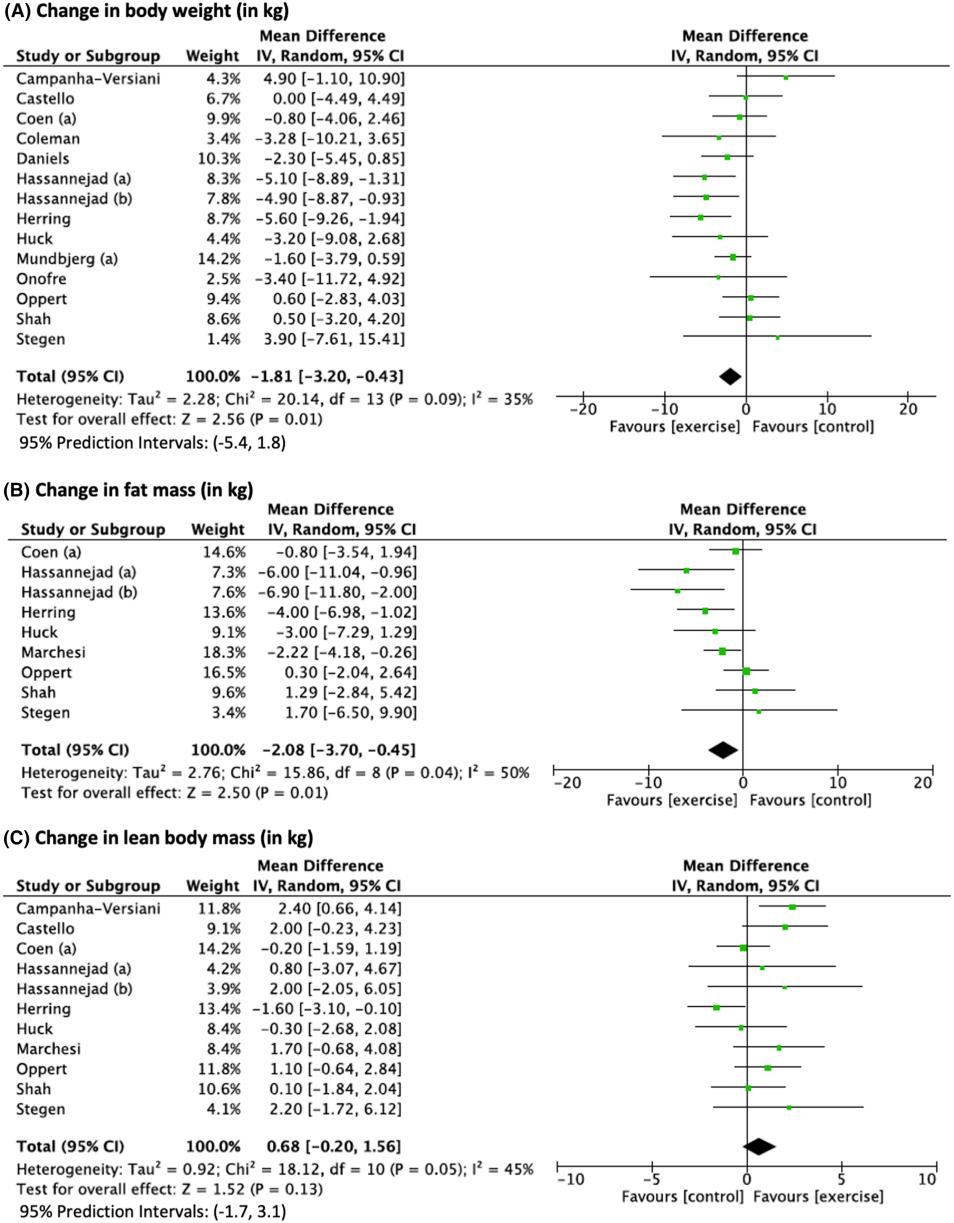
Changes in body weight (A), fat mass (B), and lean body mass (C) after bariatric surgery in exercise group compared with control group. Presents the difference in change in body weight and body composition after bariatric surgery between the participants in the exercise and control groups. Exercise training was performed after surgery in all studies. Hassannejad et al. (a)^17^: aerobic training. Hassannejad et al. (b)^17^: aerobic and resistance training. Mundbjerg et al. (a),^27^ Coen et al. (a)^26^

A greater decrease in body weight (*N* = 14 studies) and fat mass (*N* = 9 studies) was observed in the exercise versus control group (Figure 2A,B), but no significant difference in lean body mass was observed (*N* = 11 studies, Figure 2C). Sensitivity analyses did not show any impact of removing poor‐quality studies on the overall effect (Table S3). Similarly, for all outcomes except for the change in lean body mass, the one‐study removed procedure did not show any impact on the overall effect (Table S4). The change in lean body mass became significant when deleting the study by Herring et al.^49^ and between‐study heterogeneity became null (MD: 0.9 [0.3; 1.6] kg, *P* = 0.007, *I* ^2^ = 0%, Tau^2^ = 0.0, *P* = 0.44). When excluding the three interventions performed during the second postoperative year,^19^, ^40^, ^49^ the change in lean body mass was significant with low heterogeneity (MD: 1.0 [0.2; 1.9] kg, *P* = 0.02, *I* ^2^ = 11%, Tau^2^ = 0.16, *P* = 0.34). Visual inspection of the funnel plots (Figure S1) suggested little evidence of publication bias, which was suggested by Egger's test (*P* = 0.22 and *P* = 0.40 for weight loss and lean mass loss outcomes, respectively).

#### 3.3.2 Physical fitness

**FIGURE 3:**
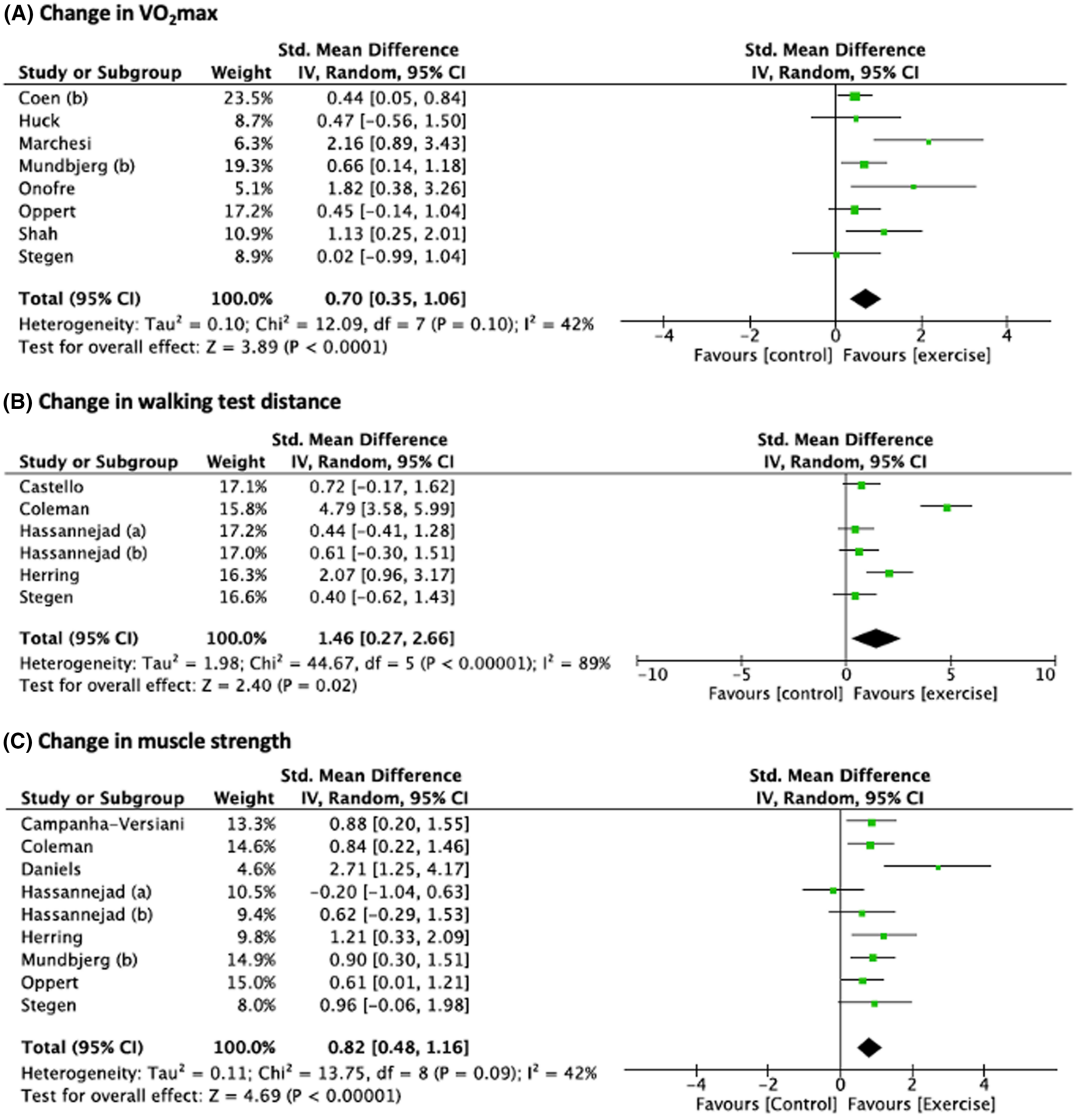
Changes in VO_2_max (A), walking test distance (B), and muscle strength (C) after bariatric surgery in exercise group compared to control group. Presents the difference in change in body weight and body composition after bariatric surgery between the participants in the exercise and control groups. Exercise training was performed after surgery in all studies. Hassannejad et al. (a)^17^: aerobic training. Hassannejad et al. (b)^17^: aerobic and resistance training. Mundbjerg et al. (b),^32^ Coen et al. (b)^28^

A greater increase in VO_2_max (eight studies, Figure 3A), walking test distance (six studies, Figure 3B), and muscle strength (nine studies, Figure 3C) was observed in the exercise group versus control group. Subsample analyses were performed on VO_2_max expressed relative to body weight. An MD of 2.73 [0.81; 4.64] mL/kg/min, *P* = 0.005, *I* ^2^ = 79%, Tau^2^ = 0.28, *P* = 0.06, *N* = 6 studies, was found. Sensitivity analyses did not show any impact on the overall effect (Tables S3 and S4), and visual inspection of the funnel plots suggested little evidence of publication bias (Figure S1).

#### 3.3.3 Bone mineral density, cardiometabolic markers, physical activity, and health‐related quality of life

A lower decrease in bone mineral density was found in the exercise group compared with the control group (*N* = 3 studies, Figure S2). No significant effect of exercise training programs was found on systolic and diastolic blood pressure (*N* = 4 studies each, Figures S4 and S6); glucose metabolism as assessed by HOMA‐IR (*N* = 2 studies, Figure S8); lipid profile as assessed by LDL‐c (*N* = 3 studies, Figure S9); HDL‐c and triglycerides (*N* = 4 studies each, Figures S11 and S13); MVPA, physical, or mental dimension of quality of life (*N* = 2 studies each, Figures S15–S18). Funnels plots are presented in Figures S3, S5, S7, S10, S12, S14, S16.

#### 3.3.4 Maintenance of effects

The maintenance of effects after a follow‐up without exercise training was assessed in two studies (with a follow‐up duration of 3 and 12 months).^27^, ^49^ At the follow‐up assessment, compared with preintervention values, a significantly greater weight loss was observed in the exercise versus control group (*N* = 2 studies, Figure S18), but no significant difference in muscle strength was found (*N* = 2 studies, Figure S19).

### 3.4 Study quality

Study quality was rated as good, fair, and poor in nine (43%),^15^, ^26^, ^27^, ^37^, ^44^, ^45^, ^48^, ^49^, ^50^ four (19%),^17^, ^18^, ^20^, ^47^ and eight (38%)^16^, ^19^, ^33^, ^36^, ^40^, ^41^, ^42^, ^43^, ^46^ studies, respectively (Table S5). Fourteen (67%), 12 (57%), and 14 (67%) studies were randomized, reported a dropout rate <20%, and reported ITT analyses, respectively. The majority of studies report high adherence, did not provide justification for sample size, and did not blind treatment assignment and outcome assessors.

## 4 DISCUSSION

This systematic review and meta‐analysis provided an updated overview on the effectiveness of exercise training in patients with obesity undergoing bariatric surgery. A novel finding of our review was that exercise training leads to a large increase in muscle strength (SMD: 0.82 [0.48; 1.16]) after bariatric surgery compared with a nonexercise control group. Gains in lower‐limb muscle strength compared with presurgery, ranging from +12%^45^ to +36%,^48^ are likely to have a beneficial impact on physical function given the major contribution of muscle strength in performing daily living activities in persons with obesity.^51^ We also found that the gain in muscle strength may be sustained after a 3‐ to 12‐month period without exercise training, although only a trend was found (*P* = 0.08), and only two studies were included in this specific meta‐analysis.

Importantly, gains in muscle strength occurred in a context of massive lean body mass loss. Even though exercise performed during the first postoperative year was found to prevent lean mass loss by 1 (95% CI: −0.2 to 1.6) kg on average in our meta‐analysis, it is far from preventing the total loss of lean mass during the same period of time (e.g., approximately 10 kg on average in the first 12 months after gastric bypass surgery).^52^ The PI was also large (−1.7 to 3.4 kg), suggesting that future studies are likely to report no significant effect of exercise of lean body mass. This loss of lean body mass may have detrimental consequences on metabolism and physical function, especially as patients get older, and may represent a risk factor for obesity sarcopenia itself associated with frailty and increased morbidity and mortality.^53^ Two hypotheses can be proposed to explain the relatively modest effect of exercise on lean body mass after bariatric surgery. First, dietary protein intake is very low in the first months after surgery and does not cover basal protein requirements.^54^ When patients were provided with protein supplementation in the form of whey protein powder, total protein intake (0.6–0.9 g/kg/day, 3‐ and 6‐month postsurgery, respectively)^45^ met the basal protein requirements but was below the amount usually recommended during resistance training (i.e., >1 g/kg/day).^54^, ^55^ Second, program duration ranged from 3 to 4.5 months in most of the included studies,^17^, ^20^, ^43^, ^45^, ^48^, ^49^ which may be insufficient to observe an effect of lean body mass although it was sufficient to observe an increase in muscle strength. In contrast, the only two studies assessing a 9‐^16^ and 24‐month^50^ training program reported a significant preservation of lean mass compared with the control group. Taken together, these findings suggest that resistance training programs performed after bariatric surgery can lead to rapid gains in muscle strength (e.g., after a 3‐month program), whereas longer interventions (e.g., >9 months) may be needed to reduce lean mass loss.

In line with previous reviews,^7^, ^12^ we also found that patients participating in an exercise training program after surgery experienced a greater improvement in cardiorespiratory fitness assessed by indirect calorimetry (VO_2_max) or by a walking test. Compared with the nonexercising control group, the mean improvement in VO_2_max in the exercise group was +2.7 (95% CI: 0.81; 4.64) mL/kg/min (SMD: 0.70 [0.35; 1.10]). This increase is slightly lower than the 3.9 mL/kg/min increase reported in our sister systematic review and meta‐analysis on the effect of exercise training in adults with obesity by van Baak et al.^56^ It was, however, observed in parallel with a large improvement in walking capacity (SMD: 1.46 [0.27; 2.66]). The two studies reporting the highest increases in VO_2_max incorporated high‐intensity periods (up to 85%–90% of HR_max_) into moderate‐intensity aerobic training.^18^, ^40^ Another recent study (see Table S6 for details) reported a significant increase in VO_2_max above the average improvement observed in our meta‐analysis (+3.4 mL/kg/min) after a 5‐month program based on aerobic training and HIIT.^57^ The number of participants was however very limited (six to 10 participants in the intervention groups), and in one study,^40^ participants were aged <50 years with a BMI < 35 kg/m^2^ and therefore may not be representative of the patients undergoing bariatric surgery. Importantly, exercise training started 3, 12, and 36 months after surgery in these three studies.^18^, ^40^, ^57^ These findings suggest that increasing the intensity of aerobic training above the moderate‐intensity threshold is feasible from 3 months after bariatric surgery and may be beneficial for further improving cardiorespiratory fitness. Previous studies conducted in adults with severe obesity (≥35 kg/m^2^) have however reported a lower adherence to an exercise program combining aerobic training with HIIT compared with aerobic training alone.^58^ No significant effect on habitual physical activity was seen in both groups.^58^ In practice, as previously recommended, intensity could be increased gradually under supervision in a pain‐free range to prevent any injury.^59^

Findings of this review show that patients participating in an exercise training program after bariatric surgery experience greater weight and fat loss by approximately 2 kg (95% CI for weight loss: −3.2; −0.4 kg). The PIs for weight loss ranged from −5.4 to 1.8 kg, suggesting that future studies are likely to report a greater weight loss in the exercise group, although it will not be the case in all settings.^60^ The mean effect is in line with two previous reviews^7^, ^8^ but in contrast with another one that reported no effect on weight loss.^9^ The latter review is specific in that it included exercise interventions based on respiratory training that is not expected to substantially increase energy expenditure.^9^ This amount of additional weight loss may be considered a relatively modest benefit compared with the 30%–35% loss of initial body weight after bariatric surgery itself.^1^ However, it is consistent with the additional weight loss described when adding exercise training during dietary weight loss interventions.^61^ Surprisingly, only two studies provided a follow‐up assessment of body weight 3 and 12 months after the end of the intervention.^27^, ^49^ The meta‐analysis of these two reviews showed a lower weight regain in the exercise group, with an MD between groups of 4.7 kg [95% CI: −7.2; −2.1 kg]. Although these findings need to be confirmed by further well‐designed trials, they do suggest that exercise may play an important role in weight maintenance after bariatric surgery. Given the prevalence of weight regain in this context (44% patients regain ≥ 5 BMI—points up to 5 years after maximal weight loss is obtained^62^), this may represent a major benefit of exercise in the clinical management of patients undergoing bariatric surgery.

This review also assessed the effect of postoperative exercise training on bone and cardiometabolic health. A meta‐analysis of three studies showed a lower decrease in bone mineral density after an exercise training program including both aerobic and resistance training. A more recent study reported similar findings.^63^ This is likely to be an important benefit of exercise training given the increased risk of fracture that has been reported after bariatric surgery, especially after gastric bypass surgery.^64^ The effects of exercise on cardiometabolic health are less conclusive in this setting. No significant effect was found on blood pressure, lipide profile, and a marker of glycemic status (i.e., HOMA‐IR). However, the number of studies included in these meta‐analyses was very limited (two to four studies for a given outcome), and findings should be interpreted with caution. Interestingly, the study by Coen et al.^26^ that included 128 patients and used gold‐standard methods to assess glucose homeostasis showed that aerobic training improves insulin sensitivity beyond the effects of bariatric surgery alone. A more recent study reported similar findings with combined aerobic and resistance training.^65^ In both studies, weight and fat loss were similar in the exercise and the nonexercise control group, suggesting an effect of exercise on insulin sensitivity independent of weight loss.

Surprisingly, we found no additional benefit of exercise on health‐related quality of life after bariatric surgery. These meta‐analyses included only two studies, but all studies reported similar findings, whether the quality of life was assessed with generic questionnaires^19^, ^33^, ^40^, ^45^, ^50^ (i.e., SF‐36 health survey) or questionnaires specific to overweight and obesity^19^ (i.e., Impact of Weight on Quality of Life, IWQOL) or to bariatric surgery^40^ (i.e., Bariatric Surgery Satisfaction Questionnaire, BSSQ). These findings contrast with the known benefits of exercise training on quality of life in adults with obesity (see our sister review by Carraça et al.).^66^ The context of bariatric surgery, however, is specific in that bariatric surgery itself is associated with marked improvement in quality of life. Changes in quality of life appear to be related to the phases of weight loss and weight regain, with peak improvements observed 6‐ to 12‐months postsurgery followed by a progressive decline in parallel with gradual weight regain.^67^ Therefore, exercise appears to have little impact on quality of life during the weight loss phase after bariatric surgery, but its effect during the weight regain phase has not yet been investigated. Similarly, we found no significant effect of exercise training programs on accelerometry‐assessed habitual physical activity. Although six studies assessed the change in physical activity in this context, we were able to include only two studies in the meta‐analysis. Original studies reported mixed findings, with most studies reporting no significant effect^15^, ^33^, ^45^, ^49^ but other studies reporting an increase^19^, ^49^ or even a decrease^30^ in accelerometry‐assessed physical activity. Including an objective assessment of habitual physical activity is of major importance to identify interventions that are effective to promote an active lifestyle outside exercise training sessions.

Preoperative interventions have received considerably less attention, with only four distinct interventions included in our review.^37^, ^41^, ^42^, ^47^ In the short term, participating in exercise training appears to improve walking capacity and may also induce a moderate weight loss, which is consistent with general findings on the effects of exercise in patients with obesity.^37^, ^41^, ^47^ A recent pilot study also suggested that a 1‐month preoperative exercise training program based on moderate‐ to high‐intensity walking may decrease the length of hospital stay.^68^ Interestingly, a larger increase in VO_2_max after the exercise training program was associated with a shorter length of hospital stay.^68^ The study by Baillot et al. was the only study providing a follow‐up assessment after surgery.^37^, ^38^ Compared with the control group, participants who had exercised during 3 months before surgery experienced a greater BMI loss and a greater increase in accelerometry‐assessed habitual physical activity 1 year after surgery.^38^ Although promising, these findings are insufficient to draw conclusions on the effectiveness of preoperative exercise training programs on long‐term weight loss outcomes after bariatric surgery.

## 5 LIMITATIONS

Although this systematic review and meta‐analysis has methodological strengths, some limitations should be mentioned. The systematic search was performed by one reviewer, and the search terms used were text words and not MeSH terms. Therefore, studies may have been missed during study selection.^69^ The number of good‐quality studies was limited, and because of heterogeneity in the interventions conducted (in terms of duration, timing, and type of exercise or intensity), important research questions could not be addressed. Of importance, we identified only two studies that directly compared different types of exercise training^17^, ^70^ of which one was published after the completion of our review.^70^ Both studies compared aerobic with combined aerobic and resistance training. They reported a slightly greater weight and fat loss and improvements in muscle strength after combined aerobic and resistance training. Regarding walking capacity, one study reported similar effects in both groups,^17^ and the other one reported higher effects with combined training.^70^ Overall, these findings suggest the superiority of combined aerobic and resistance training but need to be confirmed by studies with larger samples.

## 6 CONCLUSION

Exercise training programs performed after bariatric surgery are effective to increase cardiorespiratory fitness and muscle strength and to optimize weight and fat loss. Preliminary evidence also suggests that exercise may reduce bone loss and prevent weight regain after surgery. The effect of preoperative exercise training programs on postsurgery outcomes has been assessed in only one study, reporting a higher weight loss in the exercise group. Although we were not able to compare the effect of different types of exercise training, programs combining aerobic and resistance training appear to be the most promising to improve both cardiorespiratory and muscular fitness and are usually recommended in the context of bariatric surgery.^71^ Also, incorporating high‐intensity bouts into continuous moderate‐intensity aerobic training may further improve cardiorespiratory fitness. Exercise is clearly an effective strategy to optimize follow‐up care after bariatric surgery, but its role in the long‐term management of patients needs to be better understood.

## AUTHOR CONTRIBUTIONS

A. B. and J. M. O. performed the literature search, study selection, data extraction, and quality assessment. A. B. performed the meta‐analysis under the supervision of J. M. O. All authors participated to the interpretation of data. A. B. and J. M. O. drafted the manuscript, and authors critically revised the manuscript.

## CONFLICT OF INTERESTS

The authors have no conflict of interest to declare.

## Supporting information

**Table S1.** Keywords included in database search strategy
Table S2. Findings of included controlled trials
Table S3. Sensitivity analyses with inclusion of good‐ and fair‐quality studies
Table S4. Sensitivity analyses with one‐study‐removed procedure
Table S5. Summary of quality assessment of controlled trials
Table S6. Characteristics and main findings of articles published between November 2019 and March 2021
Figure S1. Funnel plot of pre‐to post‐surgery change in body weight (A), fat mass (B), lean body mass (C), VO^2^max (D), walking distance (E) and muscle strength (F)
Figure S2. Meta‐analysis of change in bone mineral density after bariatric surgery in exercise group compared to control group
Figure S3. Funnel plot of pre‐ to post‐surgery change in bone mineral density
Figure S4. Meta‐analysis of change in systolic blood pressure after bariatric surgery in exercise group compared to control group
Figure S5. Funnel plot of pre‐ to post‐surgery change in systolic blood pressure
Figure S6. Meta‐analysis of change in diastolic blood pressure after bariatric surgery in exercise group compared to control group
Figure S7. Funnel plot of pre‐ to post‐surgery change in diastolic blood pressure
Figure S8. Meta‐analysis of change in HOMA‐IR after bariatric surgery in exercise group compared to control group
Figure S9. Meta‐analysis of change in LDL‐cholesterol after bariatric surgery in exercise group compared to control group
Figure S10. Funnel plot of pre‐ to post‐surgery change in LDL‐cholesterol
Figure S11. Meta‐analysis of change in HDL‐cholesterol after bariatric surgery in exercise group compared to control group
Figure S12. Funnel plot of pre‐ to post‐surgery change in HDL‐cholesterol
Figure S13. Meta‐analysis of change in triglycerides after bariatric surgery in exercise group compared to control group
Figure S14. Funnel plot of pre‐ to post‐surgery change in triglycerides
Figure S15. Meta‐analysis of change in MVPA after bariatric surgery in exercise group compared to control group
Figure S16. Funnel plot of pre‐ to post‐surgery change in MVPA
Figure S17. Meta‐analysis of change in physical dimension of quality of life after bariatric surgery in exercise group compared to control group
Figure S18. Meta‐analysis of change in mental dimension of quality of life after bariatric surgery in exercise group compared to control group
Figure S19. Meta‐analysis of change in body weight after bariatric surgery in exercise group compared to control group, after a follow‐up without exercise
