# Supplementary material for: Effect of exercise training before and after bariatric surgery: A systematic review and meta‐analysis
Source: Obes Rev. 2021 Jun 3;22(Suppl 4):e13296. doi: 10.1111/obr.13296 (PMC8365633; doi:10.1111/obr.13296)
Supplement: Supplementary file 1 — Table S1. Keywords included in database search strategy Table S2. Findings of included controlled trials Table S3. Sensitivity analyses with inclusion of good‐ and fair‐quality studies Table S4. Sensitivity analyses with one‐study‐removed procedure Table S5. Summary of quality assessment of controlled trials Table S6. Characteristics and main findings of articles published between November 2019 and March 2021 Figure S1. Funnel plot of pre‐to post‐surgery change in body weight (A), fat mass (B), lean body mass (C), VO2max (D), walking distance (E) and muscle strength (F) Figure S2. Meta‐analysis of change in bone mineral density after bariatric surgery in exercise group compared to control group Figure S3. Funnel plot of pre‐ to post‐surgery change in bone mineral density Figure S4. Meta‐analysis of change in systolic blood pressure after bariatric surgery in exercise group compared to control group Figure S5. Funnel plot of pre‐ to post‐surgery change in systolic blood pressure Figure S6. Meta‐analysis of change in diastolic blood pressure after bariatric surgery in exercise group compared to control group Figure S7. Funnel plot of pre‐ to post‐surgery change in diastolic blood pressure Figure S8. Meta‐analysis of change in HOMA‐IR after bariatric surgery in exercise group compared to control group Figure S9. Meta‐analysis of change in LDL‐cholesterol after bariatric surgery in exercise group compared to control group Figure S10. Funnel plot of pre‐ to post‐surgery change in LDL‐cholesterol Figure S11. Meta‐analysis of change in HDL‐cholesterol after bariatric surgery in exercise group compared to control group Figure S12. Funnel plot of pre‐ to post‐surgery change in HDL‐cholesterol Figure S13. Meta‐analysis of change in triglycerides after bariatric surgery in exercise group compared to control group Figure S14. Funnel plot of pre‐ to post‐surgery change in triglycerides Figure S15. Meta‐analysis of change in MVPA after bariatric surgery in exercise group compared [file OBR-22-e13296-s001.pdf]

SUPPORTING INFORMATION

Effect of exercise training before and after bariatric surgery: A systematic review and meta-analysis

Alice Bellicha*[1,2], Marleen A. van Baak[3], Francesca Battista[4], Kristine Beaulieu[5], John E. Blundell[5], Luca Busetto[6,7], Eliana V. Carraça[8], Dror Dicker[6,9], Jorge Encantado[10], Andrea Ermolao[4], Nathalie Farpour-Lambert[6,11], Adriyan Pramono[3], Euan Woodward[6], Jean-Michel Oppert[12]

*Corresponding Author

Alice Bellicha, PhD

Service de Nutrition, Hôpital Pitié-Salpêtrière

47-83 Boulevard de l'Hôpital

75013 PARIS, France

Tel: +33(1)42175782

Fax:+33(1)42175963

E-mail: alice.bellicha@u-pec.fr

Table S1. Keywords included in database search strategy

Physical activity	Age	Bariatric surgery
Physical activit*	NOT child, children, adolescents, pediatric)	Bariatric surgery
Exercise		Obesity surgery
Sport		Metabolic surgery
Endurance activit*		Roux-en-Y
Aerobic activit*		Gastric bypass
Cardiovascular activit*		Sleeve gastrectomy
Resistance training		Gastric banding
Strength training		Duodenal switch
Muscle-strengthening		Post-bariatric
Weight-Lifting program		
High-intensity interval training		
HIIT		
Physical conditioning		
Walking		
Sedentary time		
Sedentary lifestyle		
Sitting time		

(Physical activit* OR Exercise OR Sport OR Endurance activit* OR Aerobic activit* OR Cardiovascular activit* OR Resistance training OR Strength training OR Muscle-strengthening OR Weight-lifting program OR High-intensity interval training OR HIIT OR Physical conditioning OR Walking OR Sedentary time OR Sedentary lifestyle OR Sitting time)
AND (Bariatric surgery OR Obesity surgery OR Metabolic surgery OR Roux-en-Y OR Gastric bypass OR Sleeve gastrectomy OR Gastric banding OR Duodenal switch OR post-bariatric)
NOT (Children OR Adolescents OR Pediatric).

Table S2. Findings of included controlled trials

Reference	Findings			Study author's conclusion	Overview authors' assessment of conclusions
	**Outcome**	**Before**	**After**		
**Preoperative interventions**					
Baillot 2016 [1]	Post-intervention			*"Adding a pre-surgical exercise training to an individual lifestyle counselling intervention improved physical fitness (…)"*	Appropriate conclusions based on available data.<br>Limited sample size
	BMI (kg/m^2)[1]	EX: 45.3 (43.7;53.5)<br>CON: 47.8 (40.3;54.0)	EX: 45.5 (43.3;53.3)<br>CON: Not reported		
	Body fat (%)[1]	EX: 49.5 (45.7;52.7)<br>CON: 49.6 (46.0;51.5)	EX: 49.8 (46.7;53.7)*<br>CON: Not reported		
	VO$_2$max (METs)[1]	EX: 7.4 (5.4;10.3)<br>CON: 6.6 (5.0;8.9)	EX: 8.4 (6.3;12.6)*<br>CON: Not reported		
	**6MWTD (m)**[1]	**EX: 489.0 (418.8;582.3)**<br>CON: 475.5 (412.0;520.0)	**EX: 502.0 (466.5;576.5) ¥**<br>CON: Not reported		
	Sit-to-stand test (rep, n)[1]	EX: 13.0 (11.5;14.0)<br>CON: 13.5 (8.8;19.3)	EX: 16.0 (16.0;18.5)*<br>CON: Not reported		
	**Arm curl test (rep, n)**[1]	**EX: 18.0 (15.3;22.5)**<br>CON: 22.0 (19.8;25.3)	**EX: 25.0 (20.3;29.0)*¥**<br>CON: Not reported		
	Quality of life[1]	EX: Not reported<br>CON: Not reported	EX: Not reported<br>CON: Not reported		
	Systolic BP (mmHg)[1]	EX: 122.0 (111.9;134.0)<br>CON: 118.3 (112.0;124.9)	EX: 123.8 (106.3;128.9)<br>CON: Not reported		
	Diastolic BP (mmHg)[1]	EX: 74.8 (66.1;80.6)<br>CON: 75.8 (70.0;85.1)	EX: 73.0 (61.8;79.8)<br>CON: Not reported		
Baillot 2017 [2]	1-year post-surgery			*"The addition of the PreSET to individual lifestyle counselling seems effective to improve PA level and submaximal physical fitness 1 year after BS"*	Appropriate conclusions based on available data except for change in submaximal physical fitness that was not significant.<br>Limited sample size.
	**BMI (kg/m^2)**	**EX: Not reported**<br>CON: Not reported	**EX: -16.8 (4.4) ¥**<br>CON: -13.5 (5.3)		
	Body fat (%)	EX: 49.3 (5.5)<br>CON: 49.1 (4.8)	EX: 33.4 (10.9)<br>CON: 35.4 (10.2)		
	**Fat-free mass (kg)**	**EX: 63.5 (12.2)**<br>CON: 65.6 (11.1)	**EX: -10.6 (4.3) ¥**<br>CON: -6.6 (3.9)		
	VO$_2$max (METs)	EX: 7.5 (2.7)<br>CON:6.8 (3.1)	EX: 11.8 (3.2)<br>CON:9.8 (3.3)		
	**6MWTD (m)**	**EX: Not reported**<br>CON: Not reported	**EX: +97.8 (56.8) ¥**<br>CON: +46.9 (70.3)		
	Sit-to-stand test (rep, n)	EX: 13.8 (4.0)<br>CON: 12.5 (6.4)	EX: 19.3 (4.0)<br>CON: 16.8 (5.5)		
	Arm curl test (rep, n)	EX: 19.6 (4.3)	EX: 24.8 (4.9)		

		CON: 22.3 (4.1)	CON: 24.3 (6.1)		
	**Moderate-intensity PA (h/d)**	**EX: Not reported**	**EX: 0.6 (0.4) ¥**		
		CON: Not reported	CON: 0.3 (0.2)		
	**Steps/d**	**EX: Not reported**	**EX: 7,460 (3,869) ¥**		
		CON: Not reported	CON: 4,288 (2,249)		
	Quality of life	EX: 66.0 (15.6)	EX: 87.3 (10.4)		
		CON: 60.1 (18.8)	CON: 80.6 (18.0)		
	Systolic BP (mmHg)	EX: 125.7 (15.7)	EX: 114.6 (12.7)		
		CON: 119.7 (9.4)	CON: 113.4 (9.0)		
	Diastolic BP (mmHg)	EX: 75.7 (9.3)	EX: 69.4 (7.3)		
		CON: 76.2 (9.9)	CON: 68.4 (8.8)		
Marcon 2017 [3]	Post-intervention			*"A 4-month, twice-weekly supervised program of low-intensity physical activity that encourages individuals to adopt a more active lifestyle can positively interfere with weight loss and improvement in functional capacity and cardiometabolic parameters of morbidly obese individuals with and without the aid of support group sessions"*	Appropriate conclusions based on available data.
	**Body weight (kg)**[2]	**EX1: 131 (6.5)**	**EX1: 123.5 (6.5)*¥**		
		**EX2: 112.6 (4.6)**	**EX2: 108.5 (4.7)*¥**		
		CON: 121 (5.8)	CON: 124 (6)*		
	**Estimated VO_2peak (mL/kg/min)**[2]	**EX1: 14.9 (0.4)**	**EX1: 15.4 (0.3)*¥**		
		EX2: 14.9 (0.4)	EX2: 15.1 (0.3)		
		CON: 14.8 (0.4)	CON: 14.6 (0.4)		
	**6MWTD (m)**[2]	**EX: 435.0 (15.0)**	**EX1: 457.5 (13.6)*¥**		
		EX2: 433.9 (16.8)	EX2: 440.6 (13.0)		
		CON: 427.2 (16.0)	CON: 418.2 (18.3)		
	**Glucose (mg/dl)**[2]	**EX1: 110.2 (9.2)**	**EX1: 99.0 (6.3) ¥**		
		**EX2: 107.2 (11.1)**	**EX2: 102.6 (9.8) ¥**		
		CON: 105.0 (7.1)	CON: 116.1 (8.5)		
	**HDL-c (mg/dl)**[2]	**EX1: 40.9 (1.6)**	**EX1: 46.2 (1.9)*¥**		
		**EX2: 43.3 (2.2)**	**EX2: 47.4 (2.4)*¥**		
		CON: 43.6 (2.0)	CON: 39.8 (1.8)*		
	**TG (mg/dl)**[2]	**EX1: 153.4 (23.3)**	**EX1: 133.5 (13.7)¥**		
		**EX2: 159.5 (22.4)**	**EX2: 147.0 (22.7)*¥**		
		CON: 153.3 (22.4)	CON: 190.2 (27.2)*		
	Systolic BP (mmHg)[2]	EX1: 140.5 (3.6)	EX1: 128.2 (2.8)*		
		EX2: 131.8 (5.0)	EX2: 129.1 (3.2)		
		CON: 130.3 (2.1)	CON: 133.1 (4.1)		
	Diastolic BP (mmHg)[2]	EX1: 88.2 (1.9)	EX1: 84 (1.9)*		
		EX2: 81.2 (3.3)	EX2: 82.0 (2.9)		
		CON: 84.7 (1.8)	CON: 86.7 (1.9)		
Marc-Hernandez 2019 [4(p)]	Post-intervention			*"The implementation of an exercise program prior to bariatric surgery reduces fat mass*	Appropriate conclusions based on available data. Limited sample size.
	**Body weight (kg)**	**EX: 137.2 (36.5)**	**EX: -7.3 (5.1)*¥**		

		CON: 113.33 (8.8)	CON: -0.6 (2.2)	*and central obesity and improves cardiometabolic risk factors and quality of life, especially in the physical scales*"	
	**Fat mass (kg)**	**EX: 67.9 (17.8)**<br>CON: 57.7 (6.9)	**EX: -7.1 (4.7)*¥**<br>CON:-1.1 (1.8)		
	Fat-free mass (kg)	EX: 69.3 (19.7)<br>CON: 55.6 (3.0)	EX: -0.2 (1.4)<br>CON:0.4 (1.6)		
	VO_2peak (mL/kg/min)	EX: 16.0 (5.1)<br>CON: 15.8 (1.2)	EX: 2.2 (16.5)*<br>CON:0.8 (1.9)		
	Leg extension torque (Nm)	EX: 156.5 (54.2)<br>CON: 141.5 (31.8)	EX: +2.8 (16.5)<br>CON:+2.5 (14.9)		
	Glucose (mg/dl)	EX: 119.7 (44.3)<br>CON: 96.7 (17.9)	EX: -17.7 (25.7)<br>CON:-3.3 (11.4)		
	HbA1c (%)	EX: 6.2 (1.3)<br>CON: 6.0 (0.9)	EX: -0.4 (0.5)*<br>CON:+0.2 (0.4)		
	LDL-c (mg/dl)	EX: 107.4 (23.3)<br>CON: 89.7 (17.2)	EX: +5.5 (7.2)<br>CON:-2.9 (5.5)		
	HDL-c (mg/dl)	EX: 43.6 (6.9)<br>CON: 55.0 (20.5)	EX: +5.0 (7.2)<br>CON: -2.9 (5.5)		
	TG (mg/dl)	EX: 135.9 (34.3)<br>CON: 121.7 (49.5)	EX: -1.5 (40.8)<br>CON:-8.4 (39.9)		
	**Systolic BP (mmHg)**	**EX: 138.4 (21.9)**<br>CON: 126.2 (22.3)	**EX: -10.5 (12.7)*¥**<br>CON: +3.9 (6.2)		
	Diastolic BP (mmHg)	EX: 83.2 (10.6)<br>CON: 77.2 (7.9)	EX: -3.9 (5.2)*<br>CON: -0.4 (5.0)		
	**SF-36 Physical score**	**EX: 34.9 (11.8)**<br>CON: 36.0 (8.5)	**EX: +7.7 (6.4)*¥**<br>CON:+1.6 (2.6)		
	SF-36 Mental score	EX: 53.1 (11.3)<br>CON: 48.1 (10.1)	EX: +1.7 (6.8)<br>CON:+3.2 (7.5)		
Pico-Sirvent 2019 [5]	Body **weight** (kg)	EX: 114.4 (19.1)<br>CON: 109.2 (10.6)	EX: 103.9 (14.8)<br>CON: 109.9 (11.4)	"*A six-month exercise training program seems to be a positive tool to improve body composition, cardiometabolic health, and fitness level in patients awaiting BS, but a larger sample size is needed to confirm these findings*"	Extremely limited sample size.
	Fat mass (kg)	EX: 50.5 (10.9)<br>CON: 53.3 (7.5)	EX: 42.2 (12.5)<br>CON: 52.9 (7.9)		
	Fat-free mass (kg)	EX: 64.0 (13.8)<br>CON: 56.0 (3.8)	EX: 61.7 (14.4)<br>CON: 57.0 (4.4)		
	VO_2peak (L/min)	EX: 2.6 (1.1)<br>CON: 1.8 (1.9)	EX: 2.7 (1.0)<br>CON: 1.9 (0.4)		
	Leg extension torque (Nm)	EX: 151.8 (36.1)<br>CON: 144.1 (21.5)	EX: 155.2 (43.4)<br>CON: 149.5 (18.4)		
	Systolic BP (mmHg)	EX: 144.2 (37.4)<br>CON: 119.6 (4.5)	EX: 139.3 (28.8)<br>CON: 120.6 (8.9)		

	Diastolic BP (mmHg)	EX: 83.2 (18.7)<br>CON: 72.7 (4.6)	EX: 79.8 (18.4)<br>CON:69.4 (6.1)		
**Post-operative interventions**					
Campanha-Versiani 2017 [6]	Body weight (kg)	EX: 109.9 ± 11.38<br>CON: 118.7 ± 20.2	EX: 73.6 ± 8.1*<br>CON: 77.5 ± 11.9*	*"The supervised exercise program attenuated lumbar spine and right hip BMD loss, improved lean mass in the arms and overall muscle strength."*	Appropriate conclusions based on available data
	Body fat (%)	EX: 48.1 ± 6.5<br>CON: 50.2 ± 6.4	EX: 33.3 ± 6.9*<br>CON: 36.0 ± 5.0*		
	**Lean body mass (kg)**	**EX: 47.0 ± 4.5**<br>CON: 47.8 ± 4.6	**EX: 44.3 ± 5.1*¥**<br>CON: 43.2 ± 3.9*		
	**Leg curls strength (kg)**	**EX: 22.0 ± 5.0**<br>CON: 21.7 ± 12.0	**EX: Not reported*¥**<br>CON: Not reported*		
	**Bone mineral density (g/cm^3)**	**EX: 1.22 ± 0.01**<br>CON: 1.24 ± 0.12	**EX: 1.15 ± 0.1*¥**<br>CON: 1.10 ± 0.01*		
Castello 2011 [7]	Body weight (kg)[2]	EX: 117.0 (4.0)<br>CON: 117.0 (6.0)	EX: 94.0 (4.0)*<br>CON: 94.0 (5.0)*	*"Aerobic exercise training improves cardiac autonomic modulation and functional capacity 4 months after GBS."*	Although the 6MWTD increased in the exercise group, the inter-group difference was not significant.<br>The conclusion made by the authors seems therefore to go beyond what the data show.
	Body fat (%)[2]	EX: 45.8 (1.4)<br>CON: 42.0 (1.5)	EX: 37.8 (1.2)*<br>CON: 36.0 (1.1)*		
	Lean body mass (kg)[2]	EX: 63.0 (3.4)<br>CON: 67.0 (1.7)	EX: 58.0 (2.9)*<br>CON: 60.0 (1.6)*		
	6MWTD (m)[2]	EX: 478 (23)<br>CON: 493 (21)	EX: 523 (18)*<br>CON: 509 (13)		
Castello-Simoes 2013 [8]	BMI (kg/m^2)[2]	EX: 45.5 (1.7)<br>CON: 46.3 (1.0)	EX: 36.5 (1.3)<br>CON: 34.2 (1.1)	*"There were no significant differences in lung function between groups"*	Appropriate conclusions based on available data
	Forced vital capacity (% predicted)[2]	EX: 94.0 (3.1)<br>CON: 99 (2.4)	EX: 101.0 (2.5)*<br>CON: 100.0 (3.7)		
Coen 2015 [9]	Body weight (kg)	EX: 108.3 (21.3)<br>CON: 106.3 (25.8)	EX: 84.3 (17.2)*<br>CON: 84.2 (21.3)*	*"Moderate exercise following RYGB surgery provides additional improvements in insulin sensitivity, glucose effectiveness, and cardiorespiratory fitness compared with a sedentary lifestyle during similar weight loss"*	Appropriate conclusions based on available data
	Fat mass (kg)	EX: 50.7 (10.3)<br>CON: 50.4 (15.2)	EX: 30.1 (10.2)*<br>CON: 31.2 (11.0)*		
	Visceral fat (cm^2)	EX: 148.8 (54.1)<br>CON: 144.8 (54.1)	EX: 81.0 (32.7)*<br>CON: 81.4 (41.0)*		
	Lean body mass (kg)	EX: 50.8 (8.2)<br>CON: 49.8 (9.6)	EX: 49.8 (7.5)<br>CON: 48.7 (9.4)*		
	**VO_2peak (ml/min)**	**EX: Not reported**<br>CON: Not reported	**EX: Not reported¥**<br>CON: Not reported*		
	**HOMA-IR (mg/dl x μIU/ml)**	**EX: 1.07 (0.51)**<br>CON: 1.37 (1.13)	**EX: 0.69 (0.31)*¥**<br>CON: 0.87 (0.58)*		
	LDL-c (mg/dl)	EX: 92.7 (25.3)<br>CON: 85.6 (23.2)	EX: 86.1 (24.1)<br>CON: 81.7 (20.3)		
	HDL-c (mg/dl)	EX: 35.7 (9.9)	EX: 47.4 (11.4)*		

		CON: 36.0 (10.9)	CON: 48.7 (10.9)*		
	TG (mg/dl)	EX: 106.3 (40.7)<br>CON: 105.3 (33.3)	EX: 80.5 (31.5)*<br>CON: 105.3 (33.3)*		
	Systolic BP (mmHg)	EX: 121.7 (13.7)<br>CON: 122.4 (14.0)	EX: 115.4 (12.1)*<br>CON: 117.0 (13.0)*		
	Diastolic BP (mmHg)	EX: 73.1 (9.4)<br>CON: 75.9 (7.9)	EX: 70.8 (8.1)<br>CON: 70.8 (8.6)*		
Coen 2015 [10]	**VO_2peak (ml/min)**<br><br>**Insulin sensitivity (µIU/ml/min)**	**EX: 1,955.3 (429.3)**<br>CON: 1,935.3 (468.1)<br>**EX: 2.27 (1.14)**<br>CON: 2.29 (1.43)	**EX: 2,068.7 (609.2)*¥**<br>CON: 1,859.8 (552.2)<br>**EX: 4.52 (2.56)*¥**<br>CON: 3.76 (2.19)*	*"Exercise superimposed on bariatric surgery–induced weight loss enhances mitochondrial respiration, induces cardiolipin remodeling, reduces specific sphingolipids, and provides additional improvements in insulin sensitivity"*	Appropriate conclusions based on available data
Woodlief 2015 [11]	Resting metabolic rate (kcal/d)	EX: Not reported<br>CON: Not reported	EX: Not reported<br>CON: Not reported	*"Resting metabolic rate decreased from 1725 (344) at randomization to 1627 (304) kcal/24 h postintervention for all subjects, but this was not different among groups."*	Appropriate conclusions based on available data
Carnero 2017 [12]	Total MVPA (min/d)<br><br>Daily steps (steps/d)<br><br>**Non-exercise PA (min/d)**<br><br>Sedentary time (min/d)	EX: 39.5 (30.2)<br>CON:42.4 (31.2)<br>EX: 5,765 (2,613)<br>CON: 6,186 (2,751)<br>**EX: Not reported**<br>CON: Not reported<br>EX: 705 (125)<br>CON: 689 (124)	EX: +14.7*<br>CON: +22.1*<br>EX: +951*<br>CON: +1,802*<br>**EX: +47.6*¥**<br>CON: +86.4*<br>EX: -59.7*<br>CON: -76.5*	*"Although the exercise program participants did not reduce NEPA, their average increase in NEPA was significantly less than those not assigned to the control group"*<br>*"Our data suggest that the similar weight and fat mass loss between RYGB and RYGB+EX could have been partially explained by NEPA"*	Appropriate conclusions based on available data
Nunez Lopez 2017 [13]	Bone mass (kg)[3]	EX: 2.8 (2.4; 3.2)<br>CON: 2.9 (2.7; 3.1)	EX : 2.7 (2.3; 3.1)*<br>CON : 2.8 (2.6; 3.0)*	*"These results indicate that the observed changes in miR-135b after the exercise intervention may contribute to bone health"*	Appropriate conclusions based on available data
Coleman 2017 [14]	<u>Post-intervention</u><br>6MWTD (m)	 EX: 495.7 (111.2)<br>CON: 503.1 (77.2)	 EX: 526.7 (87.0)*<br>CON: 503.1 (79.7)	*"When compared to patients in usual care, a specially adapted exercise program for post-*	Significance of inter-group differences is not reported. Data do not support the

	Chair rise (rep, n)	EX: 11.0 (3.5)<br>CON: 11.0 (3.5)	EX: 11.6 (4.2)<br>CON: 11.6 (3.8)	*bariatric patients resulted in significant improvements in objectively monitored health outcomes"*	conclusion made by the authors.
	Steps/d	EX: 6,633.3 (3,352.8)<br>CON: 6,640.5 (2,795.5)	EX: 7,615.3 (4,944.6)<br>CON: 6,800.7 (3,650.3)		
	6-month follow-up				
	6MWTD (m)	--	EX: 521.2 (78.6)*<br>CON: 508.0 ± 94.6		
	Chair rise (rep, n)	--	EX: 12.3 (2.6)<br>CON: 12.4 (3.6)		
	Steps/d	--	EX: 8624.9 (5436.8)<br>CON: 7409.0 (4254.7)		
Daniels 2017 [15]	Body weight (kg)	EX: 111.8 (15.3)<br>CON: 111.9 (15.2)	EX: 95.3 (13.4)<br>CON: 97.7 (12.5)	*"The resistance training intervention significantly improved muscular strength and quality; however, it did not illicit changes in FFM or muscle cross-sectional area in women who underwent Roux-en-Y gastric bypass surgery"*	Appropriate conclusions based on available data.<br>Limited sample size.
	Quadriceps CSA (cm^2)	EX: 53.8 (6.4)<br>CON: 52.3 (6.6)	EX: 53.3 (6.5)<br>CON: 50.8 (7.2)		
	**Leg press 1-RM (kg)**	**EX: 163.4 (34.4)**<br>CON: 131.1 (33.5)	**EX: 222.8 (42.4)*¥**<br>CON: 126.3 (37.9)		
Hassannejad 2017 [16]	**Body weight (kg)**	**EX1: 127.6 (18.8)**<br>**EX2: 119.7 (13.6)**<br>CON: 121.2 (25.0)	**EX1: 102.5 (18.4) ¥**<br>**EX2: 94.8 (9.7) ¥**<br>CON: 101.2 (22.7)	*"The data suggests a positive effect of exercise on weight and percent body fat decrease after surgery, and it leads to significant improvement on aerobic capacity. Moreover, doing resisted exercise caused greater preserving of lean mass"*	Appropriate conclusions based on available data.
	**Fat mass (kg)**	**EX1: 65.8 (11.4)**<br>**EX2: 57.2 (7.8)**<br>CON: 61.7 (16.4)	**EX1: 48.8 (12.7) ¥**<br>**EX2: 39.3 (7.3) ¥**<br>CON: 50.7 (14.9)		
	**Body fat (%)**	**EX1: 51.4 (2.9)**<br>**EX2: 47.9 (4.7)**<br>CON: 50.9 (6.9)	**EX1: 47.2 (5.6) ¥**<br>**EX2: 41.5 (6.5) ¥**<br>CON: 49.6 (6.2)		
	**Fat-free mass (kg)**	EX1: 61.8 (9.0)<br>**EX2: 62.4 (10.2)**<br>CON: 59.4 (14.9)	EX1: 53.7 (8.5)<br>**EX2: 55.5 (8.9) ¥**<br>CON: 50.5 (10.3)		
	**Upper limbs 1-RM (kg)**	EX1: 15.9 (7.1)<br>**EX2: 17.7 (9.4)**<br>CON: 12.5 (6.7)	EX1: 14.8 (6.2)<br>**EX2: 18.7 (9.0) ¥**<br>CON: 11.9 (7.0)		
	**12-min walk-run test**	EX1: 702.2 (185.1)<br>**EX2: 776.0 (105.1)**<br>**CON: 540.6 (1774)**	EX1: 973.8 (156.9)<br>**EX2: 1070.0 (141.7) ¥**<br>**CON: 757.5 (181.7) ¥**		
Herring 2017 [17]	Post-intervention			*"A 12-week supervised exercise intervention led to significant*	Appropriate conclusions based on available data.<br>Limited sample size.
	**Body weight (kg)**	**EX: 106.5 (16.4)**<br>CON: 106.0 (17.6)	**EX: -2.4 (3.4) ¥**<br>CON: 1 (1.4)		

	**Fat mass (kg)**	**EX: 45.2 (12.9)**<br>CON: 47.9 (10.0)	**EX: -2.1 (2.6) ¥**<br>CON: +0.9 (1.3)	*improvements in body mass and functional walking ability post intervention, with further improvements at the 24-week follow-up."*
	Fat-free mass (kg)	EX: 61.2 (9.3)<br>CON: 58.1 (12.4)	EX: -0.3 (1.4)<br>CON: +0.2 (1.3)	
	**Shuttle walk test distance (m)**	**EX: 325.0 (117.3)**<br>CON: 355.0 (80.6)	**EX: +112.5 (66.6) ¥**<br>CON: -3.3 (17.8)	
	**5 x sit-to-stand test (s)**	**EX: 13.7 (6.8)**<br>CON: 12.2 (2.9)	**EX: -3.8 (4.1) ¥**<br>CON: +0.2 (2.8)	
	**MVPA (min/d)**	**EX: 28.3 (24.0)**<br>CON: 29.7 (18.7)	**EX: +10.5 (9.2) ¥**<br>CON: -1.5 (14.5)	
	Steps/d	EX: 6379.4 (3316.0)<br>CON: 5,737.2 (1,749.4)	EX: +624 (1,350)<br>CON: +490 (1,885)	
	Sedentary time (min/d)	EX: 559.6 (94.7)<br>CON: 531.1 (131.4)	EX: -38.3 (100.0)<br>CON: -13 (69.2)	
	**Systolic BP (mmHg)**	**EX: 121.9 (16.4)**<br>CON: 120.4 (10.9)	**EX: -7.4 (11.2) ¥**<br>CON: +3.7 (8.4)	
	**Diastolic BP (mmHg)**	**EX: 80.8 (6.9)**<br>CON: 78.4 (7.7)	**EX: -5.3 (5.6) ¥**<br>CON: +3.3 (6.2)	
	<u>3-month follow-up</u>			
	**Body weight (kg)**	--	**EX: -2.7 (5.4) ¥**<br>CON: 2.9 (2.9)	
	**Fat mass (kg)**	--	**EX: -1.9 (4.1) ¥**<br>CON: 2.1 (2.8)	
	**Fat-free mass (kg)**	--	**EX: -0.8 (1.7) ¥**<br>CON: 0.8 (1.8)	
	**Shuttle walk test distance (m)**	--	**EX: +143.3 (86.6) ¥**<br>CON: -32.5 (75.9)	
	**5 x sit-to-stand test (s)**	--	**EX: -4.2 (4.0) ¥**<br>CON: 0.2 (2.1)	
	MVPA (min/d)	--	EX: +7.5 (19.8)<br>CON: -3.4 (16.2)	
	Steps/d	--	EX: +242.7 (2,358.1)<br>CON:+530.4 (2,300.2)	
	Sedentary time (min/d)	--	EX: -15.5 (89.4)<br>CON: -5.6 (79.5)	
	**Systolic BP (mmHg)**	--	**EX: -6.9 (9.2) ¥**<br>CON: 0.4 (6.7)	
	**Diastolic BP (mmHg)**	--	**EX: -5.2 (5.6) ¥**<br>CON: +2.7 (4.3)	
Huck 2015 [18]	Body weight (kg)	EX: 101.6 ± 19.8	EX: -8.8 (6.2)*	

		CON: 92.5 ± 15.5	CON: -5.6 (5.3)*	*"Supervised resistance training safely facilitates improvements in strength and physical functioning, increasing the patient's capacity to perform activities of daily living after bariatric surgery."*	Appropriate conclusions based on available data except for muscle strength that was measured only in the exercise group.<br>Limited sample size.
	Fat mass (kg)	EX: Not reported 4<br>CON: Not reported	EX: -7.0 (4.5)*<br>CON: -4.0 (3.9)*		
	Fat-free mass (kg)	EX: Not reported<br>CON: Not reported	EX: -1.8 (2.1)*<br>CON: -1.5 (2.6)		
	VO_2max (ml/kg/min)	EX: Not reported<br>CON: Not reported	EX: +0.91 (0.81)*<br>CON: +0.46 (0.97)		
	**Sit-to-stand test (%)**	**EX: Not reported**<br>CON: Not reported	**EX: +44 (15.2)*¥**<br>CON: +11.4 (23.8)		
	Systolic BP (mmHg)	EX: Not reported<br>CON: Not reported	EX: +6.9 (16.6)<br>CON: -0.25 (6.5)		
	Diastolic BP (mmHg)	EX: Not reported<br>CON: Not reported	EX: +1.4 (11.3)<br>CON: -1.8 (2.9)		
Marchesi et al. (2015) [19]	BMI (kg/m^2)[4]	EX: 29.3 (23.9; 33.6)<br>CON: 30.1 (25.9; 39.3)	EX: 27.1 (22.9; 31.7)<br>CON: 29.8 (25.2; 39.6)	*"When organized in structured and motivating protocols, aerobic physical activity seems to exert positive effects on physical and psychological health along with good compliance in small groups of carefully monitored post-bariatric patients."*	Appropriate conclusions based on available data.<br>Limited sample size.
	Fat mass (kg)[4]	EX: 27.0 (22.8; 35.8)<br>CON: 29.3 (21.1; 37.4)	EX: 24.9 (19.3; 36.1)<br>CON: 29.4 (21.0; 38.0)		
	Lean body mass (kg)[4]	EX: 50.2 (43.8; 52.3)<br>CON: 52.0 (41.8; 62.1)	EX: 51.8 (43.8; 57.5)<br>CON: 51.9 (41.3; 62.2)		
	**VO_2max (ml/kg/min)**[4]	**EX: 22.1 (18.5; 23.5)**<br>CON: 19.9 (17.2; 22.9)	**EX: 28.2 (21.6; 31.0)* ¥**<br>CON: 20.1 (17.0; 24.0)		
	Glucose (mg/dl)[4]	EX: 83 (77; 97)<br>CON: 82 (72; 92)	EX: 83 (80; 89)<br>CON: 81 (69; 89)		
	HDL-c (mg/dl)[4]	EX: 49 (43; 56)<br>CON: 49 (40; 77)	EX: 53 (38; 67)<br>CON: 46 (38; 55)		
	TG (mg/dl)[4]	EX: 85 (72; 133)<br>CON: 79 (59; 122)	EX: 78 (31; 135)<br>CON: 78 (47; 115)		
	Quality of life[4]	EX: Not reported<br>CON: Not reported	EX: Not reported<br>CON: Not reported		
Mundbjerg 2018 [20]	<u>Post-intervention</u>			*"Physical training following RYGB improves weight loss and cardiovascular health"*	Appropriate conclusions based on available data.
	Body weight (kg)	EX: 99.7 (18.0)<br>CON: 98.4 (19.3)	EX: 91.6 (18.0)<br>CON: 91.9 (18.2)		
	Abdominal fat volume (mL)	EX: 503.4 (183.9)<br>CON: 520.3 (217.4)	EX: 344.7 (131.6)<br>CON: 411.1 (220.6)		
	HbA1c (mmol/mol)	EX: 34.2 (4.1)<br>CON: 34.8 (6.3)	EX: 34.0 (4.8)<br>CON: 35.0 (5.9)		
	HOMA-IR	EX: 2.40 (1.67)<br>CON: 2.21 (1.91)	EX: 1.90 (1.19)<br>CON: 1.89 (1.42)		
	LDL (mmol/L)	EX: 2.5 (0.5)<br>CON: 2.1 (0.7)	EX: 2.4 (0.6)<br>CON: 2.2 (0.8)		

	**HDL (mmol/L)**	**EX: 1.1 (0.2)**<br>CON: 1.1 (0.2)	**EX: 1.3 (0.3) ¥**<br>CON: 1.2 (0.2)		
	Triglycerides (mmol/L)	EX: 1.0 (0.4)<br>CON: 1.0 (0.3)	EX: 0.9 (0.4)<br>CON: 0.9 (0.3)		
	<u>12-month follow-up</u>				
	**Body weight (kg)**	**--**	**EX: 91.5 (19.2) ¥**<br>CON: 94.0 (19.7)		
	Total abdominal fat volume (mL)	--	EX: 351.2 (145.7)<br>CON: 442.5 (193.8)		
	HbA1c (mmol/mol)	--	EX: 34.1 (4.1)<br>CON: 36.6 (7.5)		
	HOMA-IR	--	EX: 1.72 (1.02)<br>CON: 1.68 (0.87)		
	LDL (mmol/L)	--	EX: 2.6 (0.6)<br>CON: 2.2 (0.8)		
	HDL (mmol/L)	--	EX: 1.4 (0.3)<br>CON: 1.3 (0.3)		
	Triglycerides (mmol/L)	--	EX: 1.0 (0.4)<br>CON: 1.0 (0.4)		
Mundbjerg 2018 [21]	<u>Post-intervention</u>			*"Supervised physical training following RYGB improved VO_2max, hip MS and physical function, but the positive effects were not maintained at follow-up."*	Appropriate conclusions based on available data.
	**VO_2peak (L/min)**	**EX: 2.61 (0.77)**<br>CON: 2.43 (0.78)	**EX: 2.85 (0.82) ¥**<br>CON: 2.41 (0.80)		
	Stair-climb test (rep, n)	EX: 7.1 (1.5)<br>CON: 6.9 (1.5)	EX: 7.8 (1.5)<br>CON: 7.2 (1.6)		
	Sit-to-stand (rep, n)	EX: 15.0 (3.5)<br>CON: 16.1 (3.8)	EX: 16.1 (3.2)<br>CON: 16.8 (3.7)		
	**Hip adduction (N)**	**EX: 145.2 (36.2)**<br>CON: 137.1 (46.3)	**EX: 153.5 (40.0) ¥**<br>CON: 132.2 (47.5)		
	<u>12-month follow-up</u>				
	VO_2peak (L/min)	--	EX: 2.50 (0.78)<br>CON: 2.49 (0.79)		
	Stair-climb test (rep, n)	--	EX: 8.0 (1.7)<br>CON: 7.4 (1.9)		
	Sit-to-stand (rep, n)	--	EX: 16.6 (4.0)<br>CON: 16.9 (4.2)		
	Hip adduction (N)	--	EX: 145.9 (37.5)<br>CON: 142.7 (55.8)		
Stolberg 2018 [22]	<u>Post-intervention</u>			*"RYGB causes substantial and sustained favorable effects on markers of inflammation and*	Appropriate conclusions based on available data
	CRP (mg/L)[1]	EX: 1.58 (0.35; 3.36)<br>CON: 1.37 (0.40; 4.09)	EX: 0.52 (0.22; 1.44)*<br>CON: 0.68 (0.30; 1.04)*		

	ICAM-1 (ng/mL)[1]	EX: 243 (222; 278)<br>CON: 243 (214; 256)	EX: 230 (211; 256)<br>CON: 225 (209; 249)	*endothelial function. Supervised physical training after RYGB did not cause additional improvements"*	
	<u>12-month follow-up</u>				
	CRP (mg/L)[1]	--<br>--	EX: 0.71 (0.43; 1.05)*<br>CON: 0.67 (0.35; 1.60)*		
	ICAM-1 (ng/mL)[1]	--<br>--	EX: 237 (213; 263)<br>CON: 228 (204; 246)		
Stolberg 2018 [23]	<u>Post-intervention</u>			*"Supervised physical training intervention improves general health 24 months after RYGB and tends to improve certain domains of PA right after the intervention period, but fails to increase the patients' overall PA level over time"*	Appropriate conclusions based on available data
	MVPA (min/d)	EX: 21.0 (16.5)<br>CON: 22.1 (11.7)	EX: Not reported<br>CON: Not reported		
	Steps/d	EX: 6136 (2851)<br>CON: 6641 (2213)	EX: Not reported<br>CON: Not reported		
	Sedentary time (min/d)	EX: 616.4 (77.6)<br>CON: 612.1 (68.1)	EX: Not reported<br>CON: Not reported		
	QOL physical score	EX: 56.1 (6.6)<br>CON: 52.5 (7.6)	EX: Not reported<br>CON: Not reported		
	QOL mental score	EX: 56.1 (5.1)<br>CON: 52.9 (9.0)	EX: Not reported<br>CON: Not reported		
	<u>12-month follow-up</u>				
	MVPA (min/d)	--	EX: Not reported<br>CON: Not reported		
	Steps/d	--	EX: Not reported<br>CON: Not reported		
	Sedentary time (min/d)	--	EX: Not reported<br>CON: Not reported		
	SF-36 physical score	--	EX: Not reported<br>CON: Not reported		
	SF-36 mental score	--	EX: Not reported<br>CON: Not reported		
Stolberg 2018 [24]	<u>Post-intervention</u>			*"We observed favorable long-term reductions in markers of thrombin generation, improved fibrin clot properties and increases in fibrinolysis after RYGB. Supervised physical training after RYGB further increased fibrinolysis"*	Appropriate conclusions based on available data.
	Thrombin generation, lag time (min)[1]	EX: 3.00 (2.69-3.33)<br>CON: 3.03 (2.67-3.33)	EX: 2.83 (2.67-3.29)<br>CON: 3.00 (2.67-3.33)		
	<u>12-month follow-up</u>				
	Thrombin generation, lag time (min)[1]	--	EX: 3.08 (2.71-3.33)<br>CON: 3.03 (2.67-3.33)		
Murai 2018 [25]	Bone mineral density (g/cm^2)	**EX: 1.299 (0.096)**<br>CON: 1.270 (0.106)	**EX: Not reported*¥**<br>CON Not reported	*"Exercise mitigated bariatric surgery-induced bone loss,*	Appropriate conclusions based on available data.

				possibly through mechanisms involving suppression in bone turnover and sclerostin."	
Muschitz 2015 [26]	**Body weight (kg)**[1]	**EX: 119.6 (110.0; 129.0)**<br>CON:120.6 (110.4; 131.8)	**EX: -43.7 (-47.5; -39.1) %*¥**<br>CON: -47.4 (-49.1; -40.0) %*	*"Vitamin D loading and ongoing vitamin D, calcium, and BMI-adjusted protein supplementation in combination with physical exercise decelerates the loss of areal BMD and LBM after bariatric surgery"*	Appropriate conclusions based on available data.
	Fat mass (kg)[1]	EX: 59.0 (51.1;71.5)<br>CON: 62.4 (53.6;72.1)	EX: -54.6 (-60.7; -51.6) %*<br>CON: -59.7 (-61.0; -57.8) %*		
	**Lean body mass (kg)**[1]	**EX: 56.8 (52.8;61.5)**<br>CON: 54.5 (52.4;60.4)	**EX: -3.5 (-2.4; -4.9) %*¥**<br>CON: -12.4 (-16.1; -8.1) %*		
	**Bone mineral density (g/cm^2)**[1]	**EX: 1.275 (1.177;1.364)**<br>CON: 1.297 (1.211;1.371)	**EX: -2.2 (-2.5; -1.5) %*¥**<br>CON: -4.4 (-5.0; -3.0) **%***		
	SF-36 Physical score	EX: Not reported<br>CON: Not reported	EX: Not reported<br>CON: Not reported		
	SF-36 Mental score	EX: Not reported<br>CON: Not reported	EX: Not reported<br>CON: Not reported		
Onofre 2017 [27]	Body weight (kg)	EX: 118.4 (21.6)<br>CON: 117.6 (7.2)	EX: 87.4 (11.7)<br>CON: 90.0 (23.8)	*"Applying a physical training program to a group of obese women after 3 months of bariatric surgery could promote a significant increase in CRF only in the trained group"*	Appropriate conclusions based on available data.<br>Limited sample size.
	**VO_2peak (ml/kg/min)**	**EX: 18.8 (3.6)**<br>CON: 20.3 (4.1)	**EX: 24.7 (5.3)*¥**<br>CON: 19.3 (4.9)		
Oppert 2018 [28]	Body weight (kg)[3]	PRO: 115.7 (14.9)<br>EX: 116.7 (15.4)<br>CON: 116.3 (19.3)	PRO: -27.2 (-29.4; -25.1)*<br>EX: -27.4 (-29.9; -24.8)*<br>CON: -28.0 (-30.6; -25.4)*	*"Loss in muscle strength observed after bariatric surgery can be overcome by resistance training with additional protein intake"*	Appropriate conclusions based on available data.
	Fat mass (kg)[3]	PRO: 57.4 (10.8)<br>EX: 59.2 (10.5)<br>CON: 58.7 (11.8)	PRO: -19.8 (-21.3; -18.2)*<br>EX: -19.4 (-21.1; -17.6)*<br>CON: -19.7 (-21.5; -17.9)*		
	Lean body mass (kg)[3]	PRO: 55.9 (6.1)<br>EX: 54.8 (5.9)<br>CON: 55.6 (8.4)	PRO: -8.2 (-9.3; -7.1)*<br>EX: -7.7 (-9.0; -6.5)*<br>CON: -8.8 (-10.1; -7.5)*		
	**Lower-limb 1-RM (kg)**[3]	PRO: 174.6 (42.0)<br>**EX: 189.2 (50.4)**<br>CON: 175.7 (53.1)	PRO: -19.6 (-41.5; 2.3)*<br>**EX: +4. 7 (-19.6; 28.9)*¥**<br>CON: -30.4 (-55.8; -5.0)*		
	VO_2peak (ml/kg/min)[3]	PRO: 18.1 (3.5)<br>EX: 17.4 (4.3)<br>CON: 19.2 (4.9)	PRO: +2.5 (0.7; 4.3)*<br>EX: +4.0 (1.9; 6.1)*<br>CON: +1.8 (-0.3; 3.9)*		
	MVPA (min/d)[3]	PRO: 25.1 (17.7-41.8)<br>EX: 20.9 (15.9-33.7)<br>CON: 25.7 (14.7-33.8)	PRO: +5.7 (-1.1; 12.5)*<br>EX: +5.7 (-2.0; 13.5)*<br>CON: +9.6 (1.9; 17.3)*		
	Steps/d[3]	PRO: 6,966.5 (2,843.7)	PRO: +629 (-560; 1,819)*		

		EX: 5,638.5 (2,119.0)<br>CON: 6,573.6 (2,188.7)	EX: +1,022 (-336; 2,379)*<br>CON: +1,716 (358; 3,074)*		
	SF36 physical score[3]	PRO: 37.1 (6.6)<br>EX: 38.1 (7.5)<br>CON: 38.1 (6.5)	PRO: +9.4 (7.2;11.6)*<br>EX: +10.0 (7.4; 12.7)*<br>CON: +8.7 (6.1; 11.2)*		
	SF36 mental score[3]	PRO: 42.8 (7.4)<br>EX: 42.5 (8.5)<br>CON: 44.2 (8.6)	PRO: +8.2 (5.3; 11.2)*<br>EX: +8.9 (5.3; 12.4)*<br>CON: +5.8 (2.3; 9.3)*		
Rojhani-Shirazi 2016 [29]	BMI (kg/m^2)	EX: 40.5 (5.4)<br>CON: 44.0 (7.2)	EX: 36.9 (5.2)*<br>CON: 40.1 (6.7)*	*"Attending balance exercises program for 4 weeks can improve the balance among obese individuals undergoing sleeve gastrectomy"*	Appropriate conclusions based on available data.
	**Single leg stance, right leg (s)**	**EX: 19.4 (10.1)**<br>CON: 15.3 (14.4)	**EX: 31.5 (13.7)*¥**<br>CON: 17.9 (9.4)		
	**Get-up-and-go (s)**	**EX: 12.2 ± 2.5**<br>CON: 13.7 ± 3.1	**EX: 8.8 ± 2.1*¥**<br>CON: 12.2 ± 3.3		
Shah 2011 [30]	Body weight (kg)	EX: 110.3 (16.6)<br>CON: 101.4 (8.7)	EX: 106.1 (15.3)*<br>CON: 96.7 (6.7)	*"A high-volume exercise program is feasible in about 50% of the patients and enhances physical fitness and reduces postprandial blood glucose in bariatric surgery patients"*	Appropriate conclusions based on available data except for postprandial blood glucose that did not differ significantly from the control group.<br>Limited sample size in the control group.
	Body fat (%)	EX: 45.0 (4.4)<br>CON: 46.2 (3.6)	EX: 44.1 (5.5)<br>CON: 44.5 (5.4)		
	Lean body mass (kg)	EX: 54.9 (6.1)<br>CON: 51.1 (6.7)	EX: 54.3 (6.2)<br>CON: 50.4 (5.5)		
	**VO_2peak (ml/kg/min)**	**EX: 17.4 ± 3.3**<br>CON: 17.2 ± 1.4	**EX: 19.2 ± 4.2*¥**<br>CON: 17.1 ± 1.7		
	**Steps/d**	**EX: 4,5000**<br>CON: Not reported	**EX: 10,000*¥**<br>CON: Not reported		
	Glucose (mg/dl)[4]	EX: 91 (77;116)<br>CON: 107 (87;195)	EX: 91 (76;112)<br>CON: 102 (87;154)		
	2-h PP glucose (mg/dl)[4]	EX: 133 (96–232)<br>CON: 143 (102–369)	EX: 119 (82–149)<br>CON: 143 (81–332)		
	LDL-c (mg/dl)	EX: 112 (37)<br>CON: 94 (31)	EX: 106 (35)<br>CON: 89 (25)		
	HDL-c (mg/dl)	EX: 55 (14)<br>CON: 51 (11)	EX: 58 (12)<br>CON: 50 (9)		
	TG (mg/dl)	EX: 109 (49)<br>CON: 104 (46)	EX: 103 (45)<br>CON: 102 (31)		
	Systolic BP (mmHg)	EX: 119.6 (9.6)<br>CON: 113.5 (6.4)	EX: 117.6 (9.6)<br>CON: 116.2 (9.6)		
	Diastolic BP (mmHg)	EX: 76.1 (7.2)<br>CON: 71.5 (6.4)	EX: 72.2 (7.5)<br>CON: 73.1 (8.9)		
	SF36 physical score	EX: 52 (9)<br>CON: 49 (6)	EX: 52 (7)<br>CON: 53 (7)		

	SF36 mental score	EX: 46 (13)<br>CON: 49 (8)	EX: 55 (7)<br>CON: 49 (11)		
Stegen 2011 [31]	Body weight (kg)	EX: 130.8 (17.8)<br>CON: 126.5 (24.7)	EX: -22.7 (5.7)*<br>CON: -26.6 (14.6)*	*"A 3×/week endurance and resistance exercise program could prevent the decrease and even induce an increase in strength in most muscle groups."*	Appropriate conclusions based on available data.
	Fat mass (kg)	EX: 63.9 (14.2)<br>CON: 57.5 (14.0)	EX: -17.3 (4.6)*<br>CON: -19.0 (10.2)*		
	Fat-free mass (kg)	EX: 66.7 (9.0)<br>CON: 69.0 (13.5)	EX: -5.4 (2.6)*<br>CON: -7.6 (4.7)*		
	VO_2peak (ml/kg/min)	EX: 17.6 (3.2)<br>CON: 17.4 (4.9)	EX: 22.1 (5.1)*<br>CON: 21.8 (6.3)*		
	**Quadriceps 1-RM (kg)**	**EX: 35.5 (11.4)**<br>CON: 57.3 (28.2)	**EX: 58.0 (25.6)*¥**<br>CON: 45.9 (25.1)		
	6MWTD (m)	EX: 485.9 (28.8)<br>CON: 475.2 (58.8)	EX: 537.9 (40.6)<br>CON: 505.2 (86.8)		

Articles are presented in alphabetical order and articles reporting results from the same trial are presented together. When results of a single trial were reported in several studies, we reported results from the parent trial or from the study with the largest sample size.

Data are mean (SD) unless otherwise stated: [1]Median (25th;75th percentile). [2]Mean (SE). [3]Mean (95% CI). [4]Median (min-max).

In the colum "After", data are either post-intervention values measures or mean changes from pre- to post-intervention.

* Significant intra-group difference (P< 0.05). ¥ Significant between-group difference (P<0.05), emphasized by bold font

EX, exercise group; CON: control group; 6MWTD, 6-min walk test distance; rep, repetitions; BP, blood pressure; PA, physical activity; 1-RM, 1 repetition maximum.

In the study by Hassannejad et al. [16], EX1= aerobic training and EX2= aerobic + resistance training.

In the study by Marcon et al. [3], EX1= exercise training and EX2: exercise training + counselling

In the study by Oppert et al. [28], PRO= protein supplementation and EX= protein supplementation + resistance training

Table S3. Sensitivity analyses with inclusion of good- and fair-quality studies

	N studies	MD [95% CI]	P-value	I^2
Change in body weight	10	-2.58 (-3,90; -1.27) kg	0.0001	18% (P=0.28)
Change in fat mass	6	-2.85 (-5.09; -0,60) kg	0.01	60% (P=0.03)
Change in lean body mass	6	-0.12 (-1.10; 0.87) kg	0.82	27% (P=0.23)
Change in bone mineral density	2	0.36 [0.10; 0.62]	0.006	0% (0.40)
	**N studies**	**SMD [95% CI]**	**P-value**	I^2
Change in VO_2max	5	0.55 (0.28; 0.81)	< 0.0001	0% (P=0.47)
Change in walking test distance	4	1.94 (0.12; 3.75)	0.04	92% (P<0.0001)
Change in muscle strength	7	0.81 (0.37; 1.26)	0.003	56% (P=0.03)

Table S4. Sensitivity analyses with one-study-removed procedure

**Change in body weight**			
**Reference removed**	**MD [95% CI] kg**	**P-value**	**I^2**
Campanha-Versiani 2017 [6]	-2.09 [-3.34; -0.84]	0.001	21%
Castello 2011 [7]	-1.94 [-3.41; -0.47]	0.01	38%
Coen 2015 [9]	-1.92 [-3.44; -0.39]	0.01	39%
Coleman 2017 [14]	-1.76 [-3.21; -0.30]	0.02	40%
Daniels 2017 [15]	-1.75 [-3.29; -0.21]	0.03	40%
Hassannejad 2017a [16]	-1.51 [-2.91; -0.14]	0.03	30%
Hassannejad 2017b [16]	-1.56 [-2.96; -0.15]	0.03	32%
Herring 2017 [17]	-1.47 [-2.79; -0.15]	0.03	24%
Huck 2015 [18]	-1.74 [-3.21; -0.28]	0.02	40%
Mundbjerg 2018 [20]	-1.83 [-3.44; -0.22]	0.03	40%
Onofre 2018 [27]	-1.77 [-3.21; -0.32]	0.02	40%
Oppert 2018 [28]	-2.07 [-3.51; -0.63]	0.005	33%
Shah 2011 [30]	2.03 [-3.48; -0.58]	0.006	35%
Stegen 2011 [31]	-1.89 [-3.30; -0.49]	0.008	37%
**Change in fat mass**			
**Reference removed**	**MD [95% CI] kg**	**P-value**	**I^2**
Coen 2015 [9]	-2.32 [-4.20; -0.44]	0.02	54%
Hassannejad 2017a [16]	-1.76 [-3.37; -0.15]	0.03	47%
Hassannejad 2017b [16]	-1.66 [-3.17; -0.15]	0.03	40%
Herring 2017 [17]	-1.77 [-3.52; -0.03]	0.05	48%
Huck 2015 [18]	-2.00 [-3.79; -0.21]	0.03	55%
Marchesi 2015 [19]	-2.11 [-4.1; -0.09]	0.04	55%
Oppert 2018 [28]	-2.53 [-4.22; -0.84]	0.003	40%
Shah 2011 [30]	-2.41 [-4.08; -0.75]	0.004	45%
Stegen 2011 [31]	-2.22 [-3.90; -0.54]	0.01	54%
**Change in lean body mass**			
**Reference removed**	**MD [95% CI] kg**	**P-value**	**I^2**
Campanha-Versiani 2017 [6]	0.39 [-0.44; 1.22]	0.36	30%
Castello 2011 [7]	0.55 [-0.37; 1.47]	0.24	45%
Coen 2015 [9]	0.84 [-0.15; 1.84]	0.10	47%
Hassannejad 2017a [16]	0.69 [-0.24; 1.62]	0.15	50%
Hassannejad 2017b [16]	0.64 [-0.28; 1.55]	0.17	49%
Herring 2017 [17]	0.93 [0.26; 1.61]	0.007	0%
Huck 2015 [18]	0.79 [-0.16; 1.74]	0.10	49%
Marchesi 2015 [19]	0.60 [-0.34; 1.53]	0.21	47%
Oppert 2018 [28]	0.65 [-0.34; 1.64]	0.20	49%
Shah 2011 [30]	0.77 [-0.21; 1.76]	0.12	50%
Stegen 2011 [31]	0.62 [-0.29; 1.54]	0.18	48%
**Change in VO_2max**			
**Reference removed**	**SMD [95% CI]**	**P-value**	**I^2**
Coen 2015 [10]	0.80 [0.36; 1.25]	0.0004	45%
Huck 2015 [18]	0.74 [0.35; 1.14]	0.0002	50%
Marchesi 2015 [19]	0.57 [0.31; 0.82]	<0.0001	4%
Mundbjerg 2018 [21]	0.75 [0.30; 1.20]	0.001	50%
Onofre 2018 [27]	0.63 [0.30; 0.97]	0.0002	36%
Oppert 2018 [28]	0.78 [0.35; 1.21]	0.0003	49%
Shah 2011 [30]	0.66 [0.28; 1.03]	0.0007	44%
Stegen 2011 [31]	0.77 [0.40; 1.14]	<0.0001	44%
**Change in walking distance**			
**Reference removed**	**SMD [95% CI]**	**P-value**	**I^2**
Castello 2011 [7]	1.62 [0.15; 3.10]	0.03	91%
Coleman 2017 [14]	1.68 [0.27; 3.09]	0.02	90%
Hassannejad 2017a [16]	1.68 [0.24; 3.13]	0.02	90%
Hassannejad 2017b [16]	1.65 [0.19; 3.10]	0.03	91%

Herring 2017 [17]	1.35 [-0.04; 2.74]	0.06	90%
Stegen 2011 [31]	0.80 [0.26; 1.34]	0.004	39%
**Change in muscle strength**			
**Reference removed**	**SMD [95% CI]**	**P-value**	**I^2**
Campanha-Versiani 2017 [6]	0.82 [0.42; 1.22]	<0.0001	49%
Coleman 2017 [14]	0.83 [0.42; 1.23]	<0.0001	49%
Daniels 2017 [15]	0.74 [0.48; 0.99]	<0.0001	0%
Hassannejad 2017a [16]	0.90 [0.62; 1.19]	<0.0001	9%
Hassannejad 2017b [16]	0.85 [0.47; 1.23]	<0.0001	48%
Herring 2017 [17]	0.78 [0.41; 1.15]	<0.0001	45%
Mundbjerg 2018 [21]	0.82 [0.41; 1.22]	<0.0001	49%
Oppert 2018 [28]	0.87 [0.47; 1.26]	<0.0001	47%
Stegen 2011 [31]	0.81 [0.44; 1.19]	<0.0001	49%
**Change in bone mineral density**			
**Reference removed**	**SMD [95% CI]**	**P-value**	**I^2**
Campanha-Versiani 2017 [6]	0.36 [0.10; 0.62]	0.006	0%
Murai 2018 [25]	0.50 [0.12; 0.89]	0.01	44%
Muschitz (2015) [26]	0.59 [0.21; 0.97]	0.003	0%
**Change in systolic blood pressure**			
**Reference removed**	**MD [95% CI] mmHg**	**P-value**	**I^2**
Coen 2015 [9]	-4.2 [-12.4; 4.0]	0.32	63%
Herring 2017 [17]	-2.6 [-7.3, 2.1]	0.28	24%
Huck 2015 [18]	-5.4 [-9.5, -1.3]	0.01	18%
Shah 2011 [30]	-3.5 [-11.8, 4.9]	0.41	65%
**Change in systolic blood pressure**			
**Reference removed**	**MD [95% CI] mmHg**	**P-value**	**I^2**
Coen 2015 [9]	-4.2 [-11.2, 2.8]	0.24	63%
Herring 2017 [17]	0.8 [-3.1, 4.7]	0.68	19%
Huck 2015 [18]	-3.8 [-11.4, 3.8]	0.33	83%
Shah 2011 [30]	-1.5 [-9.1, 6.1]	0.71	83%
**Change in LDL-c**			
**Reference removed**	**SMD [95% CI]**	**P-value**	**I^2**
Coen 2015 [9]	-0.30 [-0.76, 0.15]	0.19	0%
Mundbjerg 2018 [20]	-0.10 [-0.42, 0.22]	0.55	0%
Shah 2011 [30]	-0.20 [-0.49, 0.09]	0.18	0%
**Change in HDL-c**			
**Reference removed**	**SMD [95% CI]**	**P-value**	**I^2**
Coen 2015 [9]	0.35 [-0.07, 0.76]	0.10	0%
Marchesi 2015 [19]	0.09 [-0.20, 0.37]	0.55	3%
Mundbjerg 2018 [20]	0.04 [-0.26, 0.35]	0.79	0%
Shah 2011 [30]	0.06 [-0.22, 0.34]	0.66	0%
**Change in triglycerides**			
**Reference removed**	**SMD [95% CI]**	**P-value**	**I^2**
Coen 2015 [9]	-0.09 [-0.50, 0.32]	0.66	0%
Marchesi 2015 [19]	0.03 [-0.25, 0.30]	0.84	0%
Mundbjerg 2018 [20]	0.01 [-0.30, 0.31]	0.96	0%
Shah 2011 [30]	0.03 [-0.25, 0.31]	0.85	0%

Table S5. Summary of quality assessment of controlled trials

References	Criteria														Total "Yes"	Total "No"	Total "other"	Fatal flaws	Quality rating[2]
	1	2	3	4	5	6	7	8	9	10	11	12	13	14					
Baillot (2016) [1]	Yes	Yes	Yes	No	No	Yes	Yes	Yes	No	Yes	Yes	No	Yes	Yes	10	4	0	0	**Good**
Campanha-Versiani (2017) [6]	No	NA	Yes	No	No	Yes	No	Yes	NR	Yes	Yes	No	Yes	No	6	6	2	3	**Poor**
Castello (2011) [7]	Yes	Yes	Yes	No	No	Yes	No	Yes	NR	NR	Yes	Yes	Yes	No	8	4	2	2	**Poor**
Coen (2015) [9]	Yes	Yes	Yes	No	Yes	Yes	Yes	Yes	No	Yes	Yes	No	Yes	Yes	11	3	0	0	**Good**
Coleman (2017) [14]	Yes	Yes	Yes	No	No	Yes	Yes	Yes	No	Yes	Yes	No	Yes	Yes	10	4	0	0	**Good**
Daniels (2017) [15]	Yes	Yes	No	No	No	No	Yes	Yes	NR	NR	Yes	No	Yes	Yes	7	5	2	0	**Good**
Hassannejad (2017) [16]	Yes	Yes	Yes	No	No	Yes	Yes	Yes	NR	Yes	Yes	Yes	Yes	No	10	3	1	1	**Fair**
Herring (2017) [17]	Yes	Yes	Yes	No	No	No	Yes	Yes	Yes	NR	Yes	Yes	Yes	Yes	10	3	1	0	**Good**
Huck (2015) [18]	No	NA	No	No	No	Yes	Yes	Yes	Yes	Yes	Yes	No	Yes	Yes	8	5	1	1	**Fair**
Marchesi (2015) [19]	No	NA	No	No	No	Yes	No	No	NR	NR	Yes	No	Yes	No	3	8	3	3	**Poor**
Marc-Hernandez (2019) [4(p)]	No	NA	NA	No	No	No	No	Yes	NA	Yes	Yes	No	Yes	Yes	5	6	3	2	**Poor**
Marcon (2018) [3]	Yes	Yes	Yes	No	Yes	Yes	No	Yes	Yes	Yes	Yes	No	Yes	Yes	11	3	0	1	**Fair**
Mundbjerg (2018) [20]	Yes	Yes	Yes	No	No	Yes	Yes	Yes	No	Yes	Yes	Yes	Yes	Yes	11	3	0	0	**Good**
Murai (2018) [25]	Yes	Yes	Yes	No	No	Yes	Yes	Yes	Yes	Yes	Yes	Yes	Yes	Yes	12	2	0	0	**Good**
Muschitz (2015) [26]	Yes	Yes	Yes	No	No	Yes	Yes	Yes	Yes	Yes	Yes	No	Yes	Yes	11	3	0	0	**Good**
Onofre (2017) [27]	No	NA	No	No	No	Yes	Yes	Yes	NR	NR	Yes	No	Yes	Yes	6	5	3	1	**Fair**
Oppert (2018) [28]	Yes	Yes	Yes	No	No	Yes	Yes	Yes	No	Yes	Yes	Yes	Yes	Yes	11	3	0	0	**Good**
Pico-Sirvent (2019) [5]	No	NA	NA	No	No	Yes	NA	NA	Yes	Yes	Yes	No	Yes	NA	5	4	5	3	**Poor**
Rojhani-Shirazi (2016) [29]	Yes	NR	No	No	No	Yes	NR	NR	NR	NR	Yes	No	Yes	No	4	5	5	2	**Poor**
Shah (2011) [30]	Yes	Yes	Yes	No	No	Yes	No	Yes	NR	Yes	Yes	No	Yes	Yes	9	4	1	2	**Poor**
Stegen (2011) [31]	No	No	No	No	No	No	No	Yes	NR	NR	Yes	No	Yes	No	3	8	3	3	**Poor**

Criteria for controlled trials: (1) Randomized study; (2) Adequate randomization method; (3) Treatment allocation concealment; (4) Blinding treatment assignment; (5) Blinding outcome assessors; (6) Similar baseline characteristics; (7) Drop-out rate <20%; (8) Differential drop-out rate between groups <15%; (9) High adherence; (10) Similar background treatments; (11) Valid and reliable outcome measures; (12) Sample size justification; (13) Pre-specified outcomes/subgroups; (14) All randomized participants analysed (ITT analysis).

Three criteria were defined as "fatal flaws" when not met: (1) Randomized study; (7) Drop-out rate < 20%; (14) Intention-to-Treat analysis). Study quality was defined as good, fair and poor when 0, 1 or ≥ 2 fatal flaws were identified.

Table S6. Characteristics and main findings of articles published between November 2019 and March 2021

Reference	Study design<br>Surgery type	Population	Intervention	Outcomes	Main findings
Auclair et al. [36]	RCT<br>SG, biliopancreatic diversion	<u>Exercise group</u>: N=36<br>Age: 42 (12) y<br>BMI: 46.8 (6.5) kg/m^2<br>Female: 82%<br><u>Control group</u>: N=17<br>Age: 42 (12) y<br>BMI: 44.5 (3.0) kg/m^2<br>Female: 74%	<u>Post-operative intervention</u><br>- Program duration: 3 months<br>- Start: 3 months after surgery<br>- Aerobic + resistance training<br>- 3 sessions/week: 35 min of moderate-intensity aerobic exercise + 25 min of resistance exercises (3 sets of 10-12 rep)<br>- Supervision: total (by clinical exercise specialists)	- Body weight<br>- Body composition<br>- VO_2peak<br>- Left ventricular diastolic dysfunction	Exercise vs. control group:<br>- Improved quality of life<br>- No significant change on weight and fat loss<br>- No significant change in left ventricular function
Dantas et al. [37]	RCT<br>RYGB	<u>Exercise group</u>: N=31<br>Age: 39 (7) y<br>BMI: 50.2 (7.2) kg/m^2<br>Female: 100%<br><u>Control group</u>: N=31<br>Age: 42 (7) y<br>BMI: 47.3 (8.5) kg/m^2<br>Female: 100%	<u>Post-operative intervention</u><br>Same intervention as Murai [25]<br>- Program duration: 6 months<br>- Start: 3 months after surgery<br>- Aerobic + resistance training<br>- 2 sessions/week: 30-60 min of moderate-intensity aerobic exercise + 3 sets of 8-12 rep on 7 resistance exercises<br>- Supervision: total (staff not reported)	- Body weight<br>- Body composition<br>- Glucose metabolism	Exercise vs. control group:<br>- Improved insulin sensitivity<br>- No significant effect on weight and fat loss
Diniz-Sousa et al. [33]	RCT<br>RYGB/SG	<u>Exercise group</u>: N=41<br>Age: 41.6 (10.5) y<br>BMI: 44.2 (6.8) kg/m^2<br>Female: 83%<br><u>Control group</u>: N=20<br>Age: 46.5 (8.5) y<br>BMI: 46.1 (4.2) kg/m^2<br>Female: 80%	<u>Post-operative intervention</u><br>- Programme duration: 11 months<br>- Start: 1 month after surgery<br>- Aerobic + resistance + balance training<br>- 3 sessions/week: 20 min of high-impact aerobic training + 10 min of balance training (10 min) + 2-3 sets of 4-12 rep on 8 resistance exercises<br>- Supervision: total (by exercise trainers)	- Body weight<br>- Body composition<br>- Bone mineral density<br>- Muscle strength<br>- Accelerometer-assessed PA	Exercise vs. control group:<br>- Lower decrease in bone mineral density<br>- Lower decrease in lean body mass<br>- Increase in high-impact PA (above a given accelerometry threshold)
Gilbertson et al. [34]	NRCT<br>RYGB, SG	<u>Exercise group</u>: N=7<br>Age: 45.6 (4.8) y<br>BMI: 43.9 (4.2) kg/m^2<br>Female: 100%<br><u>Control group</u>: N=7	<u>Pre-operative intervention</u><br>- Program duration: 1 month<br>- Aerobic training<br>- 5 sessions/week: 30 min of walking<br>- Supervision: none	- Body weight<br>- Body composition<br>- VO_2peak<br>- Glucose metabolism	Exercise vs. control group:<br>- No significant change in weight and fat loss, VO_2peak, insulin sensitivity<br>- Decrease in length of hospital stay

		Age: 39.0 (5.3) y<br>BMI: 46.0 (3.0) kg/m^2<br>Female: 86%			
Gilbertson et al. [35]	NRCT<br>RYGB, SG	Same participants as Gilbertson et al. [34]	Same intervention as Gilbertson et al. [34]	- Habitual PA<br>- Quality of life<br>- Blood pressure<br>- Arterial stiffness<br>- Glucose metabolism<br>- Lipid profile	Exercise vs. control group:<br>- Increase in VO_2peak<br>- No significant change in arterial stiffness, cardiometabolic markers
In et al. [32]	NRCT<br>RYGB, SG	<u>All participants:</u><br>Age: 41.0 (13.0) y<br>Female: 19%<br><u>Aerobic training group</u>: N=17<br>BMI: 41.4 (6.1) kg/m^2<br><u>Aerobic + resistance training group</u>: N=18<br>BMI: 45.0 (7.5) kg/m^2	<u>Post-operative intervention</u><br>- Program duration: 3 months<br>- Start: 1 month after surgery<br>- Aerobic or Aerobic + resistance training<br>- 3 sessions/week: 60 min of walking (aerobic group) or a combination of walking and resistance exercises with elastic bands (aerobic + resistance group)<br>- Supervision: none	- Body weight<br>- Body composition<br>- Walking test (6MWT)<br>- Muscle strength<br>- Quality of life	Aerobic + resistance training vs. aerobic training:<br>- Larger weight and fat loss<br>- Lower decrease in lean mass and bone mass<br>- No significant change in quality of life
Marc-Hernandez et al. [39]	RCT<br>SG	<u>Exercise group</u>: N=11<br>Age: 50.6 (6.6) y<br>BMI: 34.4 (7.0) kg/m^2<br>Female: 70%<br><u>Control group</u>: N=10<br>Age: 46.4 (11.2) y<br>BMI: 32.8 (4.3) kg/m^2<br>Female: 88%	<u>Post-operative intervention</u><br>- Program duration: 5 months<br>- Start: 3 years after surgery<br>- Aerobic + resistance training<br>- 2-3 sessions/week: 20 min of HIIT + 50 min of resistance exercises<br>- Supervision: total (graduates in sports sciences)	- Body weight<br>- Body composition<br>- VO_2peak<br>- Quality of life<br>- Glucose metabolism<br>- Lipid profile	Exercise vs. control group:<br>- Significant weight and fat loss, significant gain in lean body mass<br>- No significant difference quality of life
Tardif et al. [38]	RCT<br>SG, biliopancreatic diversion	<u>Exercise group</u>: N=17<br>Age: 46.4 (6.5) y<br>BMI: 50.2 (7.2) kg/m^2<br>Female: 76%<br><u>Control group</u>: N=17<br>Age: 39.3 (10.7) y<br>BMI: 45.0 (5.1) kg/m^2<br>Female: 73%	<u>Post-operative intervention</u><br>- Program duration: 3 months<br>- Start: 3 months after surgery<br>- Aerobic + resistance training<br>- 3 sessions/week: 30 min of moderate-intensity aerobic exercise + 25 min of resistance exercises<br>- Supervision: total (staff not reported)	- Body weight<br>- Body composition<br>- Lipid profile	Exercise vs. control group:<br>- Increase in HDL-c<br>- No significant difference in LDL and triglycerides<br>- No significant effect on weight, fat and lean body mass loss

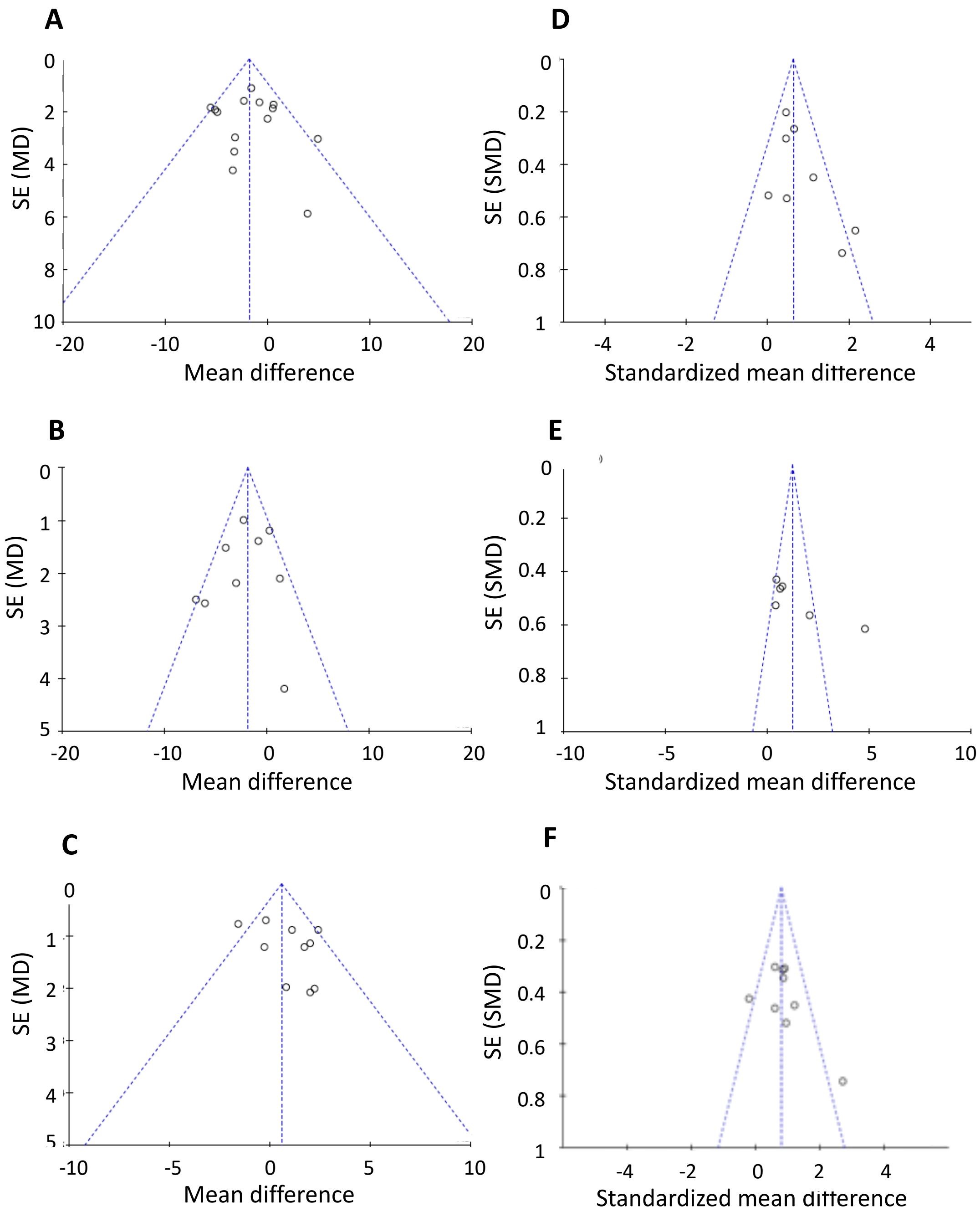

Abbreviations: error; MD, mean difference

Figure S1. Funnel plot of pre- to post-surgery change in body weight (A), fat mass (B), lean body mass (C), VO_2max (D), walking distance (E) and muscle strength (F)

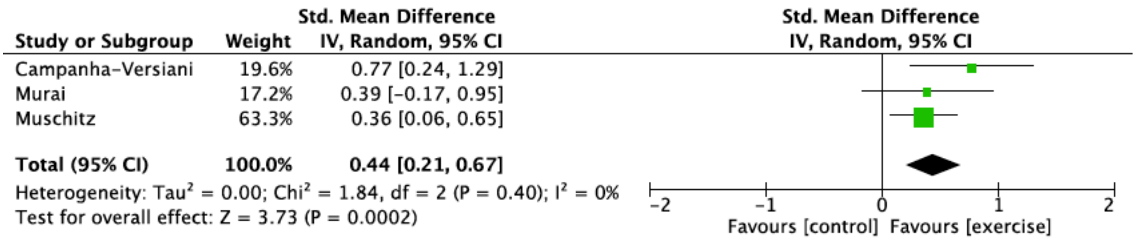

Figure S2. Meta-analysis of change in bone mineral density after bariatric surgery in exercise group compared to control group

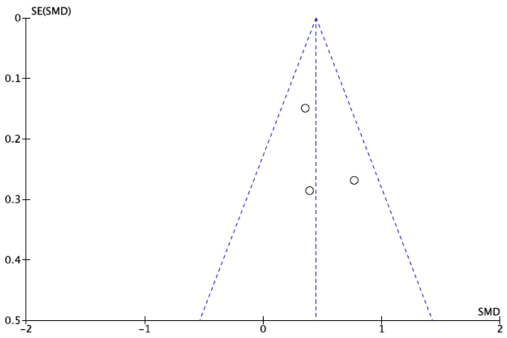

Abbreviations: error; MD, mean difference

Figure S3. Funnel plot of pre- to post-surgery change in bone mineral density

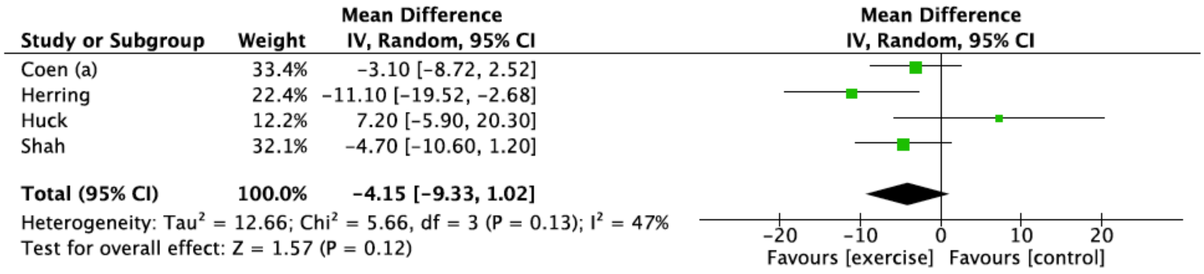

Figure S4. Meta-analysis of change in systolic blood pressure after bariatric surgery in exercise group compared to control group

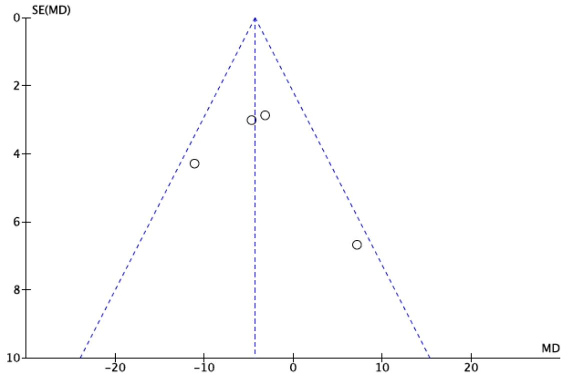

Abbreviations: error; MD, mean difference

Figure S5. Funnel plot of pre- to post-surgery change in systolic blood pressure

Study or Subgroup	Weight	Mean Difference IV, Random, 95% CI
Coen (a)	31.1%	1.80 [−1.67, 5.27]
Herring	28.0%	−8.60 [−13.67, −3.53]
Huck	20.7%	3.20 [−5.41, 11.81]
Shah	20.1%	−5.50 [−14.44, 3.44]
**Total (95% CI)**	**100.0%**	**−2.29 [−8.50, 3.91]**

Heterogeneity: $Tau^2 = 29.07$; $Chi^2 = 12.92$, df = 3 (P = 0.005); $I^2 = 77\%$
Test for overall effect: Z = 0.72 (P = 0.47)

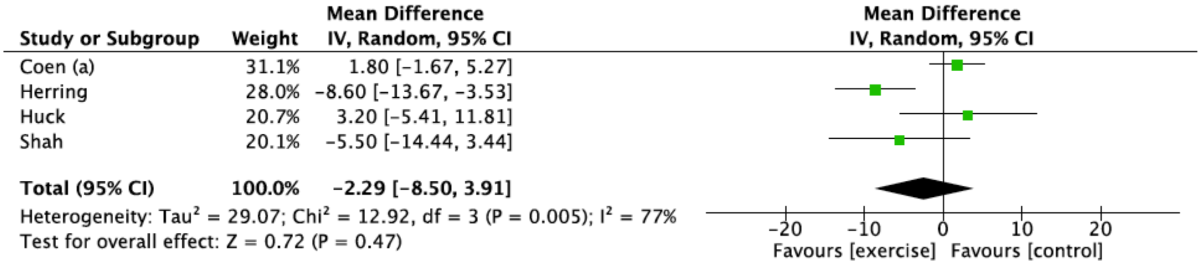

Figure S6. Meta-analysis of change in diastolic blood pressure after bariatric surgery in exercise group compared to control group

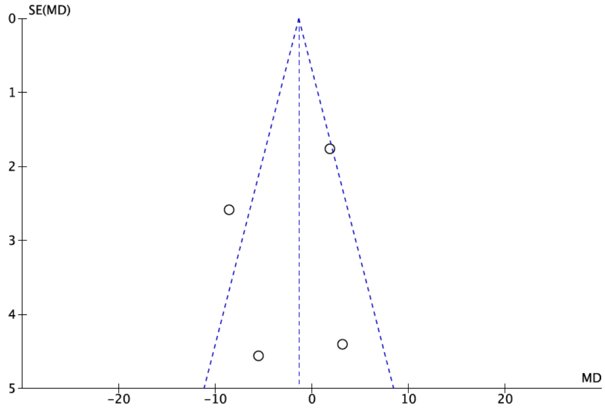

Abbreviations: error; MD, mean difference

Figure S7. Funnel plot of pre- to post-surgery change in diastolic blood pressure

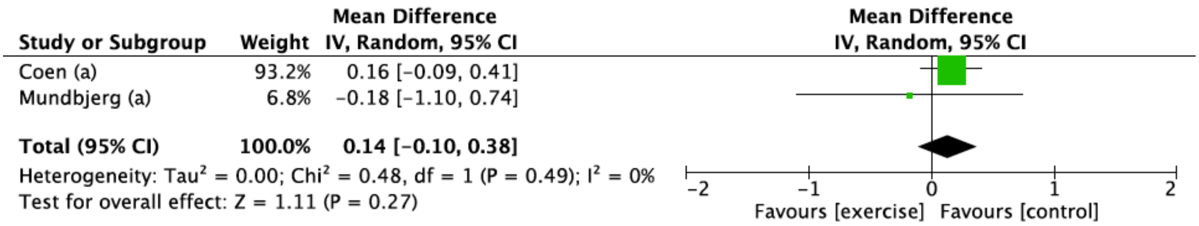

Figure S8. Meta-analysis of change in HOMA-IR after bariatric surgery in exercise group compared to control group

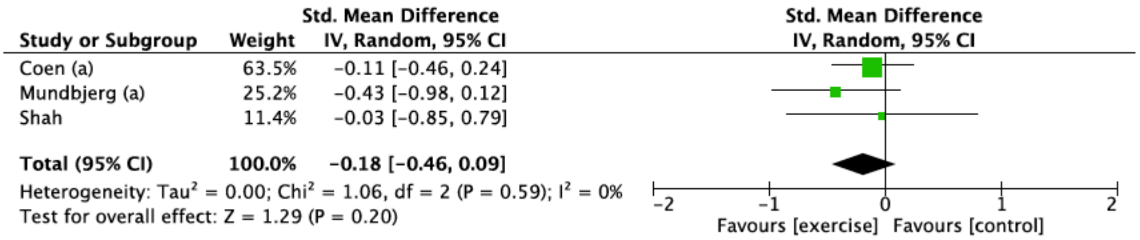

Figure S9. Meta-analysis of change in LDL-cholesterol after bariatric surgery in exercise group compared to control group

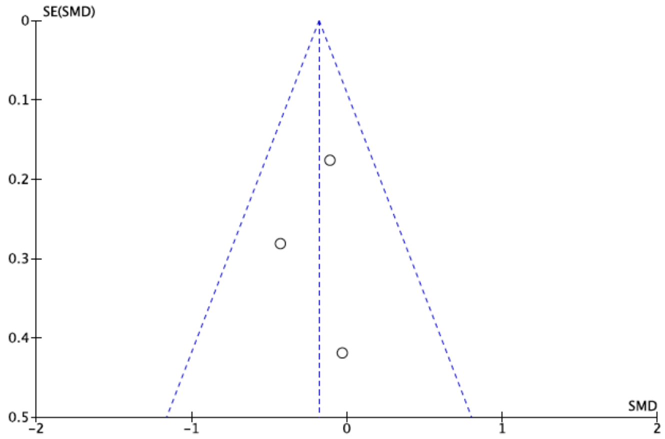

Abbreviations: error; MD, mean difference

Figure S10. Funnel plot of pre- to post-surgery change in LDL-cholesterol

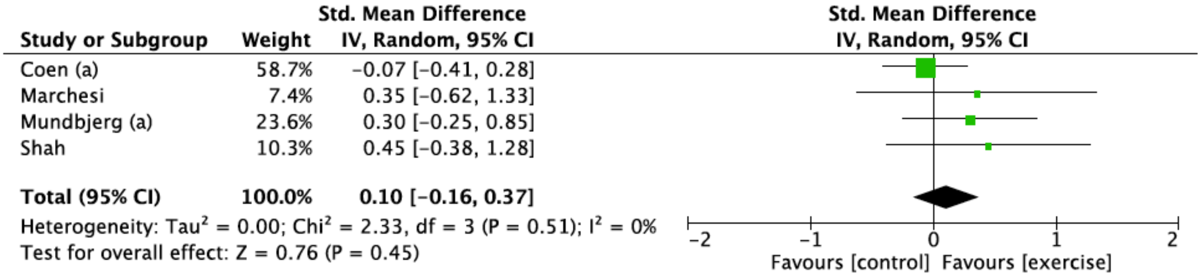

Figure S11. Meta-analysis of change in HDL-cholesterol after bariatric surgery in exercise group compared to control group

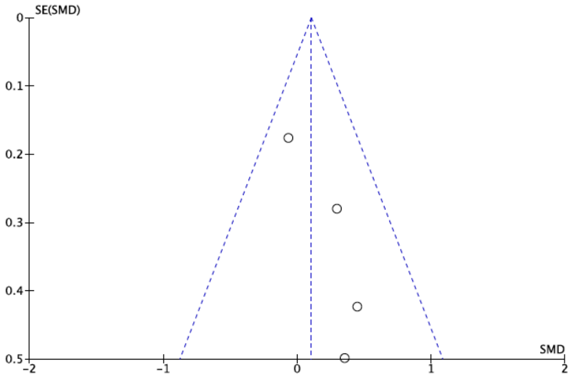

Abbreviations: error; MD, mean difference

Figure S12. Funnel plot of pre- to post-surgery change in HDL-cholesterol

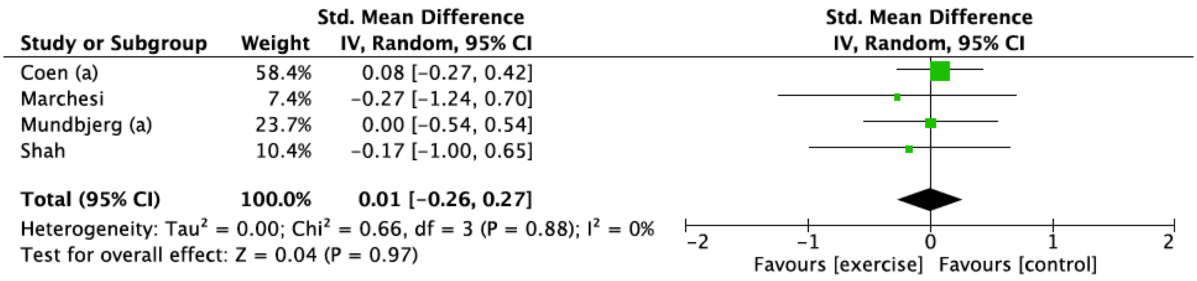

Figure S13. Meta-analysis of change in triglycerides after bariatric surgery in exercise group compared to control group

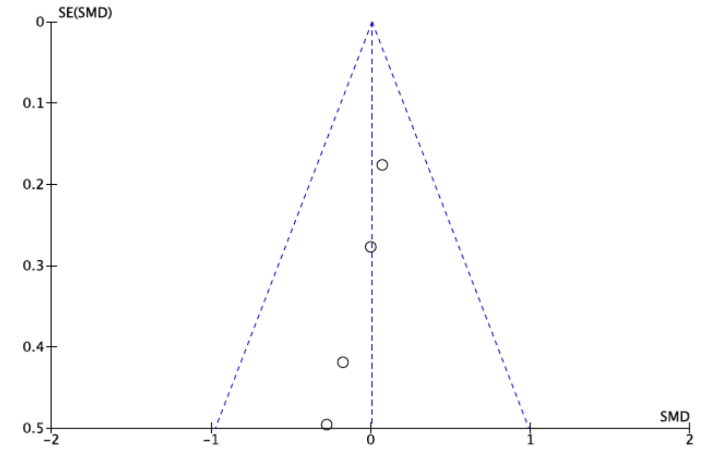

Abbreviations: error; MD, mean difference

Figure S14. Funnel plot of pre- to post-surgery change in triglycerides

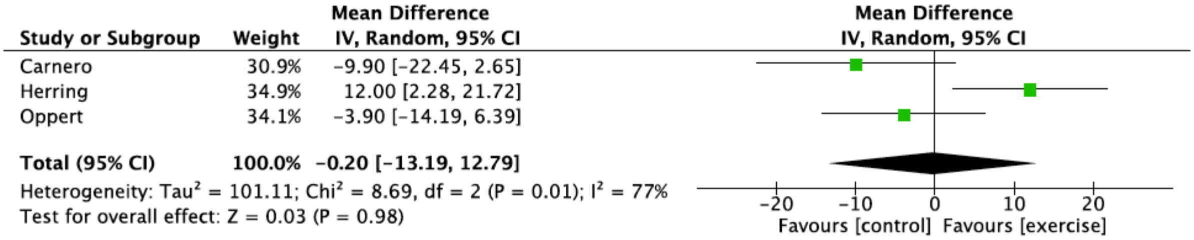

Figure S15. Meta-analysis of change in MVPA after bariatric surgery in exercise group compared to control group

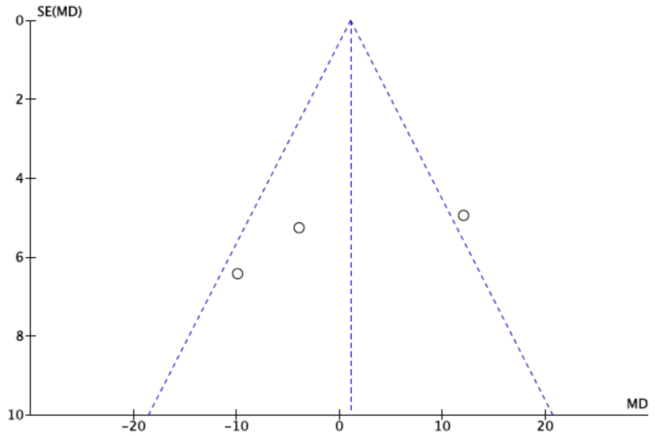

Abbreviations: error; MD, mean difference

Figure S16. Funnel plot of pre- to post-surgery change in MVPA

Study or Subgroup	Weight	Mean Difference IV, Random, 95% CI
Oppert	56.8%	−1.30 [−4.81, 2.21]
Shah	43.2%	−4.00 [−8.02, 0.02]
**Total (95% CI)**	**100.0%**	**−2.47 [−5.11, 0.17]**

Heterogeneity: $Tau^2 = 0.00$; $Chi^2 = 0.98$, df = 1 (P = 0.32); $I^2 = 0\%$
Test for overall effect: Z = 1.83 (P = 0.07)

Mean Difference
IV, Random, 95% CI

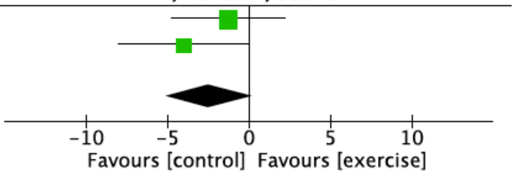

Figure S17. Meta-analysis of change in physical dimension of quality of life after bariatric surgery in exercise group compared to control group

Study or Subgroup	Weight	Mean Difference IV, Random, 95% CI	Mean Difference IV, Random, 95% CI
Shah	13.8%	9.00 [−2.89, 20.89]	
Oppert	86.2%	3.10 [−1.66, 7.86]	
**Total (95% CI)**	**100.0%**	**3.92 [−0.51, 8.34]**	

Heterogeneity: $Tau^2 = 0.00$; $Chi^2 = 0.82$, df = 1 (P = 0.37); $I^2 = 0\%$
Test for overall effect: Z = 1.74 (P = 0.08)

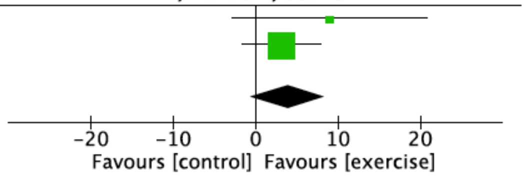

Figure S18. Meta-analysis of change in mental dimension of quality of life after bariatric surgery in exercise group compared to control group

Study or Subgroup	Exercise Mean	Exercise SD	Exercise Total	Control Mean	Control SD	Control Total	Weight
Herring	−2.7	5.4	11	2.9	2.9	10	48.8%
Mundbjerg (a)	−8.2	5.9	22	−4.4	5.9	20	51.2%
**Total (95% CI)**			**33**			**30**	**100.0%**

Heterogeneity: $Tau^2 = 0.00$; $Chi^2 = 0.48$, df = 1 (P = 0.49); $I^2 = 0\%$
Test for overall effect: Z = 3.58 (P = 0.0003)

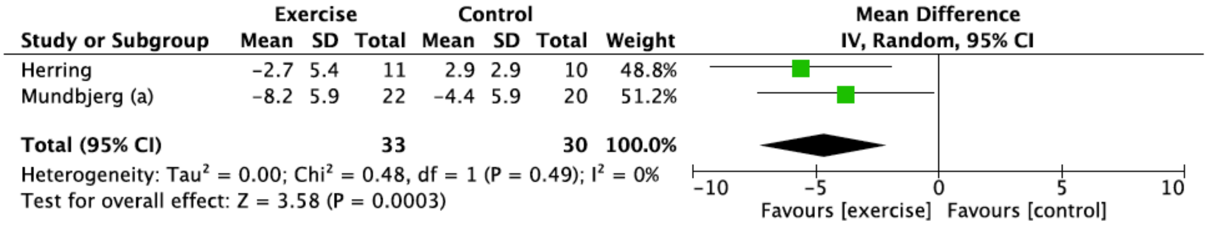

Figure S19. Meta-analysis of change in body weight after bariatric surgery in exercise group compared to control group, after a follow-up without exercise

Study or Subgroup	Weight	Std. Mean Difference IV, Random, 95% CI
Mundbjerg (b)	59.0%	0.41 [−0.20, 1.03]
Herring	41.0%	1.30 [0.34, 2.26]
**Total (95% CI)**	**100.0%**	**0.78 [−0.08, 1.64]**

Heterogeneity: $Tau^2 = 0.23$; $Chi^2 = 2.34$, df = 1 (P = 0.13); $I^2 = 57\%$
Test for overall effect: Z = 1.78 (P = 0.08)

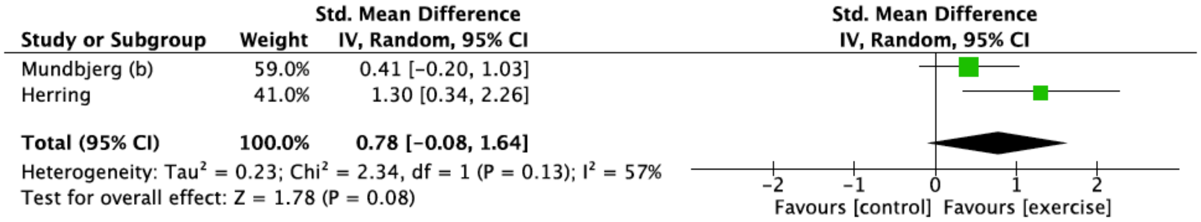

Figure S20. Meta-analysis of change in muscle strength after bariatric surgery in exercise group compared to control group, after a follow-up without exercise

References

1. Baillot A, Mampuya WM, Dionne IJ, Comeau E, Meziat-Burdin A, Langlois MF. Impacts of Supervised Exercise Training in Addition to Interdisciplinary Lifestyle Management in Subjects Awaiting Bariatric Surgery: a Randomized Controlled Study. *Obes Surg*. 2016;26(11):2602-2610. doi:10.1007/s11695-016-2153-9

2. Baillot A, Vallee CA, Mampuya WM, et al. Effects of a Pre-surgery Supervised Exercise Training 1 Year After Bariatric Surgery: a Randomized Controlled Study. *Obes Surg*. 2018;28(4):955-962. doi:10.1007/s11695-017-2943-8

3. Marcon ER, Baglioni S, Bittencourt L, Lopes CL, Neumann CR, Trindade MR. What Is the Best Treatment before Bariatric Surgery? Exercise, Exercise and Group Therapy, or Conventional Waiting: a Randomized Controlled Trial. *Obes Surg*. 2017;27(3):763-773. doi:10.1007/s11695-016-2365-z

4. Marc-Hernández A, Ruiz-Tovar J, Aracil A, Guillén S, Moya-Ramón M. Impact of Exercise on Body Composition and Cardiometabolic Risk Factors in Patients Awaiting Bariatric Surgery. *Obes Surg*. 2019;29(12):3891-3900. doi:10.1007/s11695-019-04088-9

5. Pico-Sirvent I, Aracil-Marco A, Pastor D, Moya-Ramon M. Effects of a Combined High-Intensity Interval Training and Resistance Training Program in Patients Awaiting Bariatric Surgery: A Pilot Study. *Sports Basel*. 2019;7(3). doi:10.3390/sports7030072

6. Campanha-Versiani L, Pereira DAG, Ribeiro-Samora GA, et al. The Effect of a Muscle Weight-Bearing and Aerobic Exercise Program on the Body Composition, Muscular Strength, Biochemical Markers, and Bone Mass of Obese Patients Who Have Undergone Gastric Bypass Surgery. *Obes Surg*. 2017;27(8):2129-2137. doi:10.1007/s11695-017-2618-5

7. Castello V, Simoes RP, Bassi D, Catai AM, Arena R, Borghi-Silva A. Impact of aerobic exercise training on heart rate variability and functional capacity in obese women after gastric bypass surgery. *Obes Surg*. 2011;21(11):1739-1749. doi:10.1007/s11695-010-0319-4

8. Castello-Simoes V, Polaquini Simoes R, Beltrame T, et al. Effects of aerobic exercise training on variability and heart rate kinetic during submaximal exercise after gastric bypass surgery--a randomized controlled trial. *Disabil Rehabil*. 2013;35(4):334-342. doi:10.3109/09638288.2012.694575

9. Coen PM, Tanner CJ, Helbling NL, et al. Clinical trial demonstrates exercise following bariatric surgery improves insulin sensitivity. *J Clin Invest*. 2015;125(1):248-257. doi:10.1172/JCI78016

10. Coen PM, Menshikova EV, Distefano G, et al. Exercise and Weight Loss Improve Muscle Mitochondrial Respiration, Lipid Partitioning, and Insulin Sensitivity After Gastric Bypass Surgery. *Diabetes*. 2015;64(11):3737-3750. doi:10.2337/db15-0809

11. Woodlief TL, Carnero EA, Standley RA, et al. Dose response of exercise training following roux-en-Y gastric bypass surgery: A randomized trial. *Obes Silver Spring*. 2015;23(12):2454-2461. doi:10.1002/oby.21332

12. Carnero EA, Dubis GS, Hames KC, et al. Randomized trial reveals that physical activity and

energy expenditure are associated with weight and body composition after RYGB. *Obes Silver Spring*. 2017;25(7):1206-1216. doi:10.1002/oby.21864

13. Nunez Lopez YO, Coen PM, Goodpaster BH, Seyhan AA. Gastric bypass surgery with exercise alters plasma microRNAs that predict improvements in cardiometabolic risk. *Int J Obes*. 2017;41(7):1121-1130. doi:10.1038/ijo.2017.84

14. Coleman KJ, Caparosa SL, Nichols JF, et al. Understanding the Capacity for Exercise in Post-Bariatric Patients. *Obes Surg*. 2017;27(1):51-58. doi:10.1007/s11695-016-2240-y

15. Daniels P, Burns RD, Brusseau TA, et al. Effect of a randomised 12-week resistance training programme on muscular strength, cross-sectional area and muscle quality in women having undergone Roux-en-Y gastric bypass. *J Sports Sci*. Published online 2017:1-7. doi:10.1080/02640414.2017.1322217

16. Hassannejad A, Khalaj A, Mansournia MA, Rajabian Tabesh M, Alizadeh Z. The Effect of Aerobic or Aerobic-Strength Exercise on Body Composition and Functional Capacity in Patients with BMI >/=35 after Bariatric Surgery: a Randomized Control Trial. *Obes Surg*. 2017;27(11):2792-2801. doi:10.1007/s11695-017-2717-3

17. Herring LY, Stevinson C, Carter P, et al. The effects of supervised exercise training 12-24 months after bariatric surgery on physical function and body composition: a randomised controlled trial. *Int J Obes*. 2017;41(6):909-916. doi:10.1038/ijo.2017.60

18. Huck CJ. Effects of supervised resistance training on fitness and functional strength in patients succeeding bariatric surgery. *J Strength Cond Res*. 2015;29(3):589-595. doi:10.1519/JSC.0000000000000667

19. Marchesi F, De Sario G, Reggiani V, et al. Road Running After Gastric Bypass for Morbid Obesity: Rationale and Results of a New Protocol. *Obes Surg*. 2015;25(7):1162-1170. doi:10.1007/s11695-014-1517-2

20. Mundbjerg LH, Ron Stolberg C, Cecere S, et al. Supervised Physical Training Improves Weight Loss After Roux-en-Y Gastric Bypass Surgery: A Randomized Controlled Trial. *Obes Silver Spring*. 2018;26(5):828-837. doi:10.1002/oby.22143

21. Mundbjerg LH, Stolberg CR, Bladbjerg EM, Funch-Jensen P, Juhl CB, Gram B. Effects of 6 months supervised physical training on muscle strength and aerobic capacity in patients undergoing Roux-en-Y gastric bypass surgery: a randomized controlled trial. *Clin Obes*. 2018;8(4):227-235. doi:10.1111/cob.12256

22. Stolberg CR, Mundbjerg LH, Funch-Jensen P, Gram B, Bladbjerg EM, Juhl CB. Effects of gastric bypass surgery followed by supervised physical training on inflammation and endothelial function: A randomized controlled trial. *Atherosclerosis*. 2018;273:37-44. doi:10.1016/j.atherosclerosis.2018.04.002

23. Stolberg CR, Mundbjerg LH, Bladbjerg EM, Funch-Jensen P, Gram B, Juhl CB. Physical training following gastric bypass: effects on physical activity and quality of life-a randomized controlled trial. *Qual Life Res*. 2018;27(12):3113-3122. doi:10.1007/s11136-018-1938-9

24. Stolberg CR, Mundbjerg LH, Funch-Jensen P, Gram B, Juhl CB, Bladbjerg EM. Effects of gastric bypass followed by a randomized study of physical training on markers of coagulation activation, fibrin clot properties, and fibrinolysis. *Surg Obes Relat Dis*. 2018;14(7):918-926. doi:10.1016/j.soard.2018.03.022

25. Murai IH, Roschel H, Dantas WS, et al. Exercise Mitigates Bone Loss in Women With Severe Obesity After Roux-en-Y Gastric Bypass: A Randomized Controlled Trial. *J Clin Endocrinol Metab*. 2019;104(10):4639-4650. doi:10.1210/jc.2019-00074

26. Muschitz C, Kocijan R, Haschka J, et al. The Impact of Vitamin D, Calcium, Protein Supplementation, and Physical Exercise on Bone Metabolism After Bariatric Surgery: The BABS Study. *J Bone Min Res*. 2016;31(3):672-682. doi:10.1002/jbmr.2707

27. Onofre T, Carlos R, Oliver N, et al. Effects of a Physical Activity Program on Cardiorespiratory Fitness and Pulmonary Function in Obese Women after Bariatric Surgery: a Pilot Study. *Obes Surg*. 2017;27(8):2026-2033. doi:10.1007/s11695-017-2584-y

28. Oppert J-M, Bellicha A, Roda C, et al. Resistance Training and Protein Supplementation Increase Strength After Bariatric Surgery: A Randomized Controlled Trial. *Obes Silver Spring*. 2018;26(11):1709-1720. doi:10.1002/oby.22317

29. Rojhani-Shirazi Z, Mansoriyan SA, Hosseini SV. The effect of balance training on clinical balance performance in obese patients aged 20-50 years old undergoing sleeve gastrectomy. *Eur Surg-Acta Chir Austriaca*. 2016;48(2):105-109. doi:10.1007/s10353-015-0379-8

30. Shah M, Snell PG, Rao S, et al. High-volume exercise program in obese bariatric surgery patients: a randomized, controlled trial. *Obes Silver Spring*. 2011;19(9):1826-1834. doi:10.1038/oby.2011.172

31. Stegen S, Derave W, Calders P, Van Laethem C, Pattyn P. Physical fitness in morbidly obese patients: effect of gastric bypass surgery and exercise training. *Obes Surg*. 2011;21(1):61-70. doi:10.1007/s11695-009-0045-y

32. In G, Taskin HE, Al M, et al. Comparison of 12-Week Fitness Protocols Following Bariatric Surgery: Aerobic Exercise Versus Aerobic Exercise and Progressive Resistance. *Obes Surg*. Published online 2021. doi:10.1007/s11695-020-05144-5

33. Diniz-Sousa F, Veras L, Boppre G, et al. The Effect of an Exercise Intervention Program on Bone Health After Bariatric Surgery: A Randomized Controlled Trial. *J Bone Min Res*. Published online 2020. doi:10.1002/jbmr.4213

34. Gilbertson NM, Gaitán JM, Osinski V, et al. Pre-operative aerobic exercise on metabolic health and surgical outcomes in patients receiving bariatric surgery: A pilot trial. *PloS One*. 2020;15(10):e0239130. doi:10.1371/journal.pone.0239130

35. Gilbertson NM, Eichner NZM, Khurshid M, et al. Impact of Pre-operative Aerobic Exercise on Cardiometabolic Health and Quality of Life in Patients Undergoing Bariatric Surgery. *Front Physiol*. 2020;11:1018. doi:10.3389/fphys.2020.01018

36. Auclair A, Harvey J, Leclerc J, et al. Determinants of Cardiorespiratory Fitness After Bariatric Surgery: Insights From a Randomised Controlled Trial of a Supervised Training Program. *Can J Cardiol*. 2021;37(2):251-259. doi:10.1016/j.cjca.2020.03.032

37. Dantas WS, Roschel H, Murai IH, et al. Exercise-Induced Increases in Insulin Sensitivity After Bariatric Surgery Are Mediated By Muscle Extracellular Matrix Remodeling. *Diabetes*. 2020;69(8):1675-1691. doi:10.2337/db19-1180

38. Tardif I, Auclair A, Piché M-E, et al. Impact of a 12-Week Randomized Exercise Training Program on Lipid Profile in Severely Obese Patients Following Bariatric Surgery. *Obes Surg*. 2020;30(8):3030-3036. doi:10.1007/s11695-020-04647-5

39. Marc-Hernández A., Ruiz-Tovar J., Aracil A., Guillén S., Moya-Ramón M. Effects of a High-Intensity Exercise Program on Weight Regain and Cardio-metabolic Profile after 3 Years of Bariatric Surgery: A Randomized Trial. *Sci Rep*. 2020;10(1):3123. doi:10.1038/s41598-020-60044-z
